# LXR-inverse agonism stimulates immune-mediated tumor destruction by enhancing CD8 T-cell activity in triple negative breast cancer

**DOI:** 10.1038/s41598-019-56038-1

**Published:** 2019-12-20

**Authors:** Katherine J. Carpenter, Aurore-Cecile Valfort, Nick Steinauer, Arindam Chatterjee, Suomia Abuirqeba, Shabnam Majidi, Monideepa Sengupta, Richard J. Di Paolo, Laurie P. Shornick, Jinsong Zhang, Colin A. Flaveny

**Affiliations:** 1 The Department of Pharmacology and Physiology, Saint Louis University School of Medicine, Saint Louis, MO 63104 USA; 2 The Center for Clinical Pharmacology, Saint Louis College of Pharmacy, Saint Louis, MO 63110 USA; 3 The Department of Molecular Microbiology and Immunology, Saint Louis University School of Medicine, Saint Louis, MO 63104 USA; 4 The Department of Biology, Saint Louis University, Saint Louis, MO 63103 USA; 5 The Alvin J. Siteman Cancer Center at Barnes-Jewish and Washington University School of Medicine in Saint Louis, Saint Louis, MO 63110 USA

**Keywords:** Cancer microenvironment, Immunoediting

## Abstract

Triple-negative breast cancer (TNBC) is a highly aggressive subtype that is untreatable with hormonal or HER2-targeted therapies and is also typically unresponsive to checkpoint-blockade immunotherapy. Within the tumor microenvironment dysregulated immune cell metabolism has emerged as a key mechanism of tumor immune-evasion. We have discovered that the Liver-X-Receptors (LXRα and LXRβ), nuclear receptors known to regulate lipid metabolism and tumor-immune interaction, are highly activated in TNBC tumor associated myeloid cells. We therefore theorized that inhibiting LXR would induce immune-mediated TNBC-tumor clearance. Here we show that pharmacological inhibition of LXR activity induces tumor destruction primarily through stimulation of CD8+ T-cell cytotoxic activity and mitochondrial metabolism. Our results imply that LXR inverse agonists may be a promising new class of TNBC immunotherapies.

## Introduction

Triple-negative breast cancer (TNBC), diagnosed in 15–20% of all breast cancer patients, has a more aggressive histology and poorer prognosis than that of all other breast cancer subtypes. TNBCs lack detectable expression of the estrogen receptor (ER/NR3A1-2) progesterone receptor (PR/NR3C3) and the receptor tyrosine kinase erbB-2 (HER2/ERBB2). As a result, TNBC is not treatable with currently available targeted therapies. If diagnosed early, TNBC responds well to radiotherapy and chemotherapy. However, the prognosis for patients with metastatic or recurrent disease is grim, with treatment limited primarily to palliative care^1^. Sadly, a large percentage of TNBC patients are diagnosed with late stage metastatic disease^2^. As a result, TNBC still remains a significant clinical challenge.

Due to low nutrient content and poor oxygenation, the tumor microenvironment is typically unfavorable to immune cell metabolic activity^3–8^. Effective immune response to malignant growth requires coordinated upregulation of glycolysis and mitochondrial oxidative phosphorylation as well as enhanced lipid synthesis^4,6,9,10^. Dysregulation of immune-cell glucose metabolism is a known mechanism of tumor immune evasion in TNBC^3^. Tumor cells can display high rates of glycolysis even when oxygen is abundant, termed the Warburg effect, that in addition to providing macromolecules for cell replication, plays a crucial role in directing tumor-immune interaction^11–14^. The Delgoffe group has shown that hypoxic conditions within tumors suppressed the metabolic activity of tumor-infiltrating immune cells and potentiated resistance to PD-1 treatment in melanoma models^7,15^. They also went on to show that PD-1 resistance was pharmacologically targetable using glycolysis inhibition^15^. These studies therefore successfully established that metabolic modulators may be effectively used in the clinic to improve responsiveness to immunotherapy.

In addition to Warburg metabolism, tumors display elevated lipid synthesis (lipogenesis) throughout early and late stage tumorigenesis. Using models of melanoma and hematological tumors Russo and colleagues were the first to establish a link between tumor lipid production and tumor-immune evasion. They revealed that that tumor cells produce lipid-metabolites, typically oxidized cholesterol metabolites, that suppress antigen presenting cell chemotaxis through disruption of dendritic cell (DC) CC-motif chemokine receptor-7 expression^8^. Their work also highlighted that genetic or drug-mediated disruption of cholesterol synthesis had immune-mediated anti-tumor effects^16,17^. These studies were the first to implicate the nuclear receptors, the Liver-X-Receptors α and β (NR1H3 and NR1H2), as molecular targets of these cholesterol metabolites and a central mediator of lipid-mediated immune suppression^8,16–21^. In particular pharmacological targeting of LXR-ligand production effectively overcome immunotherapy resistance in preclinical melanoma models^16,21^. LXR has also been cited as a marker for poor prognosis, particularly in African American TNBC patients^22,23^. In breast cancer overall, elevated lipid metabolism is irrevocably linked to poor clinical outcomes and treatment resistance^24–26^. Yet whether there is an LXR-dependent/lipid-mediated mechanism of immune evasion has not been elucidated for TNBC. Crucially, the efficacy of pharmacologically modulating LXR as an immunotherapeutic strategy, has not been tested in TNBC.

LXRs are ligand activated transcription factors that function as cholesterol sensors as they are activated by oxysterols; the most abundant endogenous LXR-ligands. Ligand-activation causes LXR, which forms obligate heterodimers with Retinoid-X-Receptors (RXR), to bind to LXR-response elements (LXREs) and recruit co-activators to the promoter and enhancer regions of target genes^27–30^. Target gene activation typically results in the upregulation of factors that regulate fatty acid synthesis such as fatty acid synthetase (*FASN)*^28^, sterol-regulatory element binding protein 1c (*SREBP-1c*)^31^, cholesterol export ABC-transporters-CA1/G1 (*ABCA1*, *ABCG1*)^32^ and cholesterol synthesis (*FDFT1*)^33^.

LXRs also play a major role in attenuating immune cell function through cross-talk with pro-inflammatory pathways. The role of LXR in regulating macrophage activity and promoting anti-inflammatory M2 differentiation is well documented^29,34–36^. In addition to inhibiting DC chemokine receptor expression, LXR activation is known to repress NFκB activity, suppress Th17, facilitate Treg differentiation, and disrupt Th1 and Th2 CD4+ T-cell differentiation^37–40^. We have shown previously that the LXR-inverse agonist, SR9243, which downregulates LXR activity to below basal levels, can be used to target tumor lipogenesis and Warburg metabolism to directly induce cancer cell death in a myriad of solid tumor types^14,41,42^. Therefore, LXR is an intriguing molecular target for exploring TNBC tumor-immune interaction. We theorized that we could effectively stimulate immune-mediated TNBC tumor-destruction by suppressing LXR activity in immune cells.

Through analysis of single-cell RNA-sequencing data (see ref. ^43^) we have discovered that LXR activity is upregulated in TNBC-tumor resident myeloid cells. In accord with this we show that mouse and human TNBC cells produce LXR agonists that specifically activate LXR and suppress macrophage M1 polarization and differentiation. We further demonstrate, using our LXR inhibitor SR9243, that blockade of LXR activation induces CD8 +  T-cell Interferon γ (IFNγ) and Granzyme B (GZMB) production and enhances CD8+ T-cell cytotoxicity. Mitochondrial profiling of CD8+ T-cells revealed that LXR activation represses mitochondrial metabolism and disrupts plasma membrane localization of cholesterol. Lastly, using tumor-allograft models of TNBC, we show that inhibiting LXR activity in immune cells promotes tumor destruction in a CD8+ T-cell dependent manner. This study is the first to illustrate that LXR-inverse agonists may be a useful approach for clinically targeting TNBC and implies that LXR may be an intriguing target for modulating CD8+ T-cell metabolism and anti-tumor activity.

## Results

### Tumor infiltrating macrophages and DCs display elevated LXR activity in human TNBC

**Figure 1:**
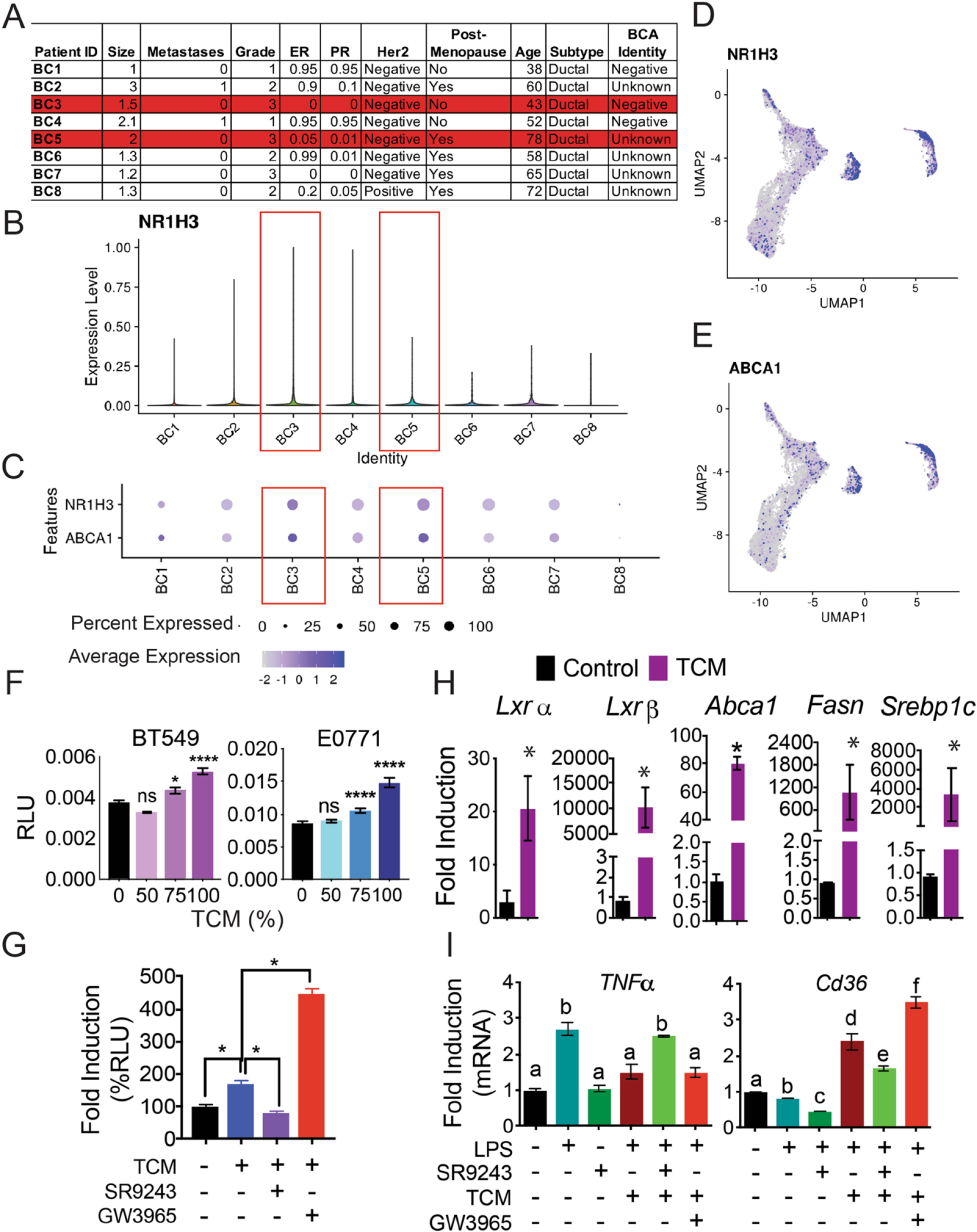
TNBC tumors produce LXR-agonists that inhibit myeloid cell activity (**A**) Table showing patient tumor pathology data. Samples BC3 and BC5 are triple-negative breast tumors. Tumor infiltrating immune cells were isolated and subjected to single cell RNA-sequencing (scRNA-seq) as described by Azizi *et al*.^43^. (**B**) scRNA-seq data showing *NR1H3* (*LXRα*) expression among tumor infiltrating immune cells from the tumors listed in A. Red boxes highlight the TNBC tumors (BC3 and BC5). (**C**) Dot-plot of sc-RNA-seq data showing the average expression and the percentage of cells expressing *LXRα* (*NR1H3*) and *ABCA1* among tumor infiltrating immune-cells. Red boxes highlight the TNBC tumors (BC3 and BC5). (**D**) sc-RNA-seq data dot-plot showing clusters of TNBC tumor-infiltrating immune cells that express *LXRα* and (**E**) *ABCA1*. Cell type clusters were generated using Seurat (see Materials and Methods section for details). (**F**) LXRE-driven Luciferase reporter assay (LXRE-Luc) showing induction of LXR transactivation in response to tumor-conditioned media (TCM). BT549: human TNBC cell line. E0771: mouse TNBC cell line. HEK-293 cells were transfected with LXRE-Luc plasmid reporter and exposed to increasing percentages of TCM for 24 h. (**G**) LXRE-Luc reporter assay showing fold induction of LXR activity in response to E0771 TCM alone or in combination with 100 nM of the LXR inverse agonist SR9243 or 100 nM GW3965. (**H**) RT-QPCR analysis showing induction of *Lxrα*, *Lxrβ*, and the *Lxr* target genes *Abca1*, *Fasn* and *Srebp1c* in bone marrow derived macrophages (BMDMs) treated with TCM or control E0771 culture media (RPMI1640 + 10%FBS). (**I**) Expression of the proinflammatory (M1) macrophage marker; *Tnfα*, or the anti-inflammatory (M2) marker; *Cd36* in LPS-activated mature BMDMs in response to 10 μM SR9243 or 5 μM GW3965 treatment for 24 h. Letters above bars identify means that are significantly different based on p < 0.05 as determined by 2-Way ANOVA.

Oxysterol-induced immune suppression, through activation of LXR in DCs and neutrophils, is a known mechanism of tumor-immune evasion^8,17–20,44^. However, it is unknown whether this mechanism of immune evasion is conserved in TNBC. We therefore examined previously published single-cell RNA-sequencing (sc-RNA-seq) data (see ref. ^43^) which comprehensively characterized tumor infiltrating immune cells, peripheral blood (PMBCs), lymph nodes and matched normal tissues isolated from patients with diverse breast cancer subtypes (Fig. 1A and Supplementary Fig. S1A). Using Seurat analysis of this scRNA-seq dataset, we were able to elucidate that TNBC tumor-resident immune cells displayed a heightened expression of *LXRα (NR1H3*) (Fig. 1B, Supplementary Fig. S1A). Specifically, *LXRα* and the ABC-cholesterol transporter; *ABCA1*, a direct target gene of LXR, were significantly upregulated in TNBC tumor-resident immune cells (Fig. 1C, Supplementary Fig. S1B). Seurat cell-cluster analysis further revealed that *NR1H3* and *ABCA1* expression was most potently induced in “myeloid cell” clusters (clusters 8 and 20: see Supplementary Fig. S1B). In addition, *NR1H3* expression strongly overlapped with that of *ABCA1* (Fig. 1D,E) in these myeloid clusters. These findings implied that *LXRα* signaling was selectively upregulated in TNBC tumor resident myeloid cells and highlighted that LXR activation in immune cells may be relevant to tumor-immune interactions in TNBC.

### TNBC tumor cells produce LXR agonists

With our observations in patient derived samples, we theorized that TNBC tumors produced endogenous LXR ligands, cholesterol metabolites, that activate LXR signaling in immune cells. We therefore assessed the effects of TNBC-produced LXR-ligands on LXR pathway regulation in macrophages and quantified the immunomodulatory effects of these ligands on macrophage function. To accomplish this, we quantified the activity of cell-free culture media from mouse (E0771 and EMT6) and human (BT549) TNBC cells in LXRE-driven luciferase reporter assays (LXRE-Luc). Interestingly, we found that TCM from TNBC cells dose-dependently modulated LXR activity (Fig. 1F, Supplementary Fig. S1C). Curiously, TCM from the mouse EMT6 cell line had mixed activity, as it inhibited LXR activity at low concentrations, yet stimulated LXR activity at high concentrations (Supplementary Fig. S1C). Conversely, TCM from E0771 and human BT549 cells displayed potent agonist activity (Fig. 1F). In particular, TCM from E0771 cells induced LXRE-Luc expression with a potency that was comparable to that of the synthetic LXR agonist GW3965 and opposite to that of the LXR inverse agonist SR9243, a potent LXR inhibitor^14^ (Fig. 1F and Supplementary Fig. S1D,E). These results confirmed our observations in patient tumor samples that support a mechanism wherein TNBC tumors produce LXR ligands that stimulate LXR activity. Our results also highlighted that E0771 cells may be a useful tool for studying tumor immune-interactions in TNBC *in vitro* and in *in vivo*.

We tested whether LXR activation by TCM could be modulated by the synthetic LXR ligands GW3965 and SR9243. As expected SR9243 inhibited TCM-induced LXRE-Luc activity and GW3965 had the opposite effect of additively enhancing LXR activity when combined with TCM (Fig. 1G). We then quantified the effect of E0771 TCM on endogenous LXR target gene expression in macrophages. Notably, in LPS-activated macrophages, LXRα and LXRβ was substantially increased upon exposure to TCM (Fig. 1H). In addition, the LXR-target genes *Abca1*, *Fasn* and *Srebp1c* were all proportionally increased by TCM (Fig. 1H). These results confirmed that TNBC cells produced LXR agonists that stimulate endogenous LXR transcriptional activity in macrophages.

### TNBC-ligands suppress macrophage activity

LXR activation is known to repress macrophage activation and proinflammatory (M1) versus tolerogenic (M2) polarization^32,34,45–48^. We therefore tested the effect of TNBC lipids, SR9243 and GW3965 on macrophage differentiation and activity. We assessed the effect of TCM on macrophage M1 and M2 polarization by quantifying the expression of the M1 marker, TNFα, and the M2 marker, CD36 in response to GW3965 or SR9243 alone or in combination with TCM. In naïve macrophages, SR9243, like LPS, decreased expression of the M2 marker CD36, whereas GW3965 alone had no effect (Supplementary Fig. S1F). In naïve macrophages, LXR ligands did not modulate TNFα expression (Supplementary Fig. S1F). Surprisingly, in contrast to LPS control, both SR9243 and GW3965 reduced eNOS expression in naïve macrophages (Supplementary Fig. S1F). Importantly, TCM inhibited LPS-induction of TNFα expression (Fig. 1I). Conversely, SR9243 de-repressed but GW3965 additively enhanced, TCM suppression of TNFα expression (Fig. 1I). In these macrophages, expression of the M2 marker CD36 was analogoulsy suppressed by LPS, but de-repressed by TCM (Fig. 1I). TCM-induced CD36 expression was, in turn, disrupted by SR9243 and additively increased by GW3965 (Fig. 1I). This outcome suggested that E0771 cells produce LXR ligands that modulate endogenous LXR activity in macrophages and implied that TNBC-produced LXR ligands may direct macrophage M1/M2 polarization.

### SR9243 stimulates Th1 differentiation selectively preserves Th1 viability

**Figure 2:**
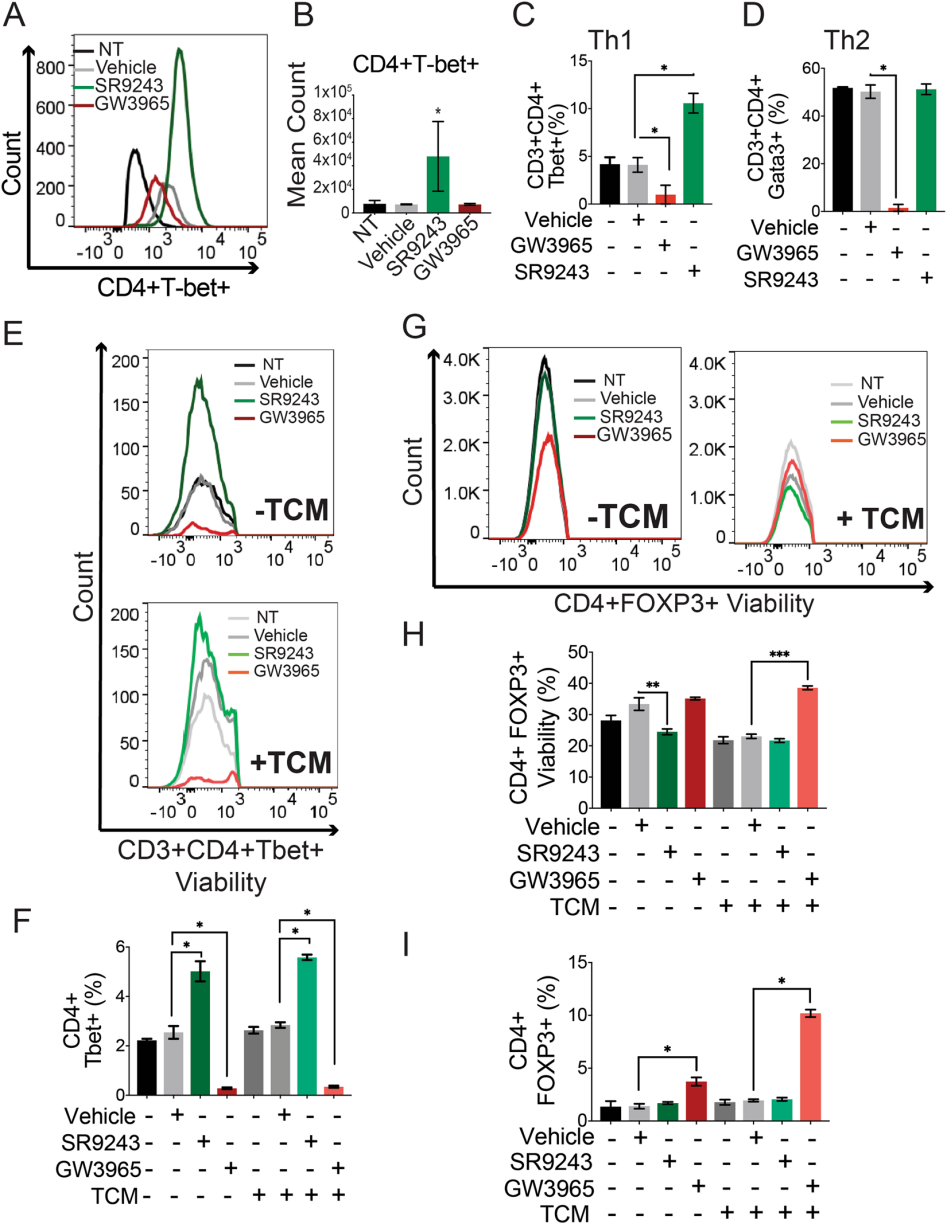
LXR inhibition using SR9243 promotes CD4+ T-cells Th1 polarization and inhibits Treg viability (**A**) FACs histogram showing Tbet expression in differentiated Th1 CD4+ T-cells (CD3+ CD4+ Tbet+) exposed to vehicle (DMSO), SR9243 or GW3965. (**B**) Mean count as determined by FACs of Th1 (Tbet+ CD3+ CD4+) T-cells generated from Th0 cells cultured in Th1 differentiation conditions (αCD3, αCD28, IL2, IL12 and αIL4) and treated with DMSO, SR9243 or GW3965 for 7 days (**C**) FACs showing the mean percentage of Th1 polarized cells generated from Th0 splenocytes cultured under Th1 polarizing conditions and exposed to DMSO vehicle, SR9243 or GW3965 treatment for 7 days. (**D**) FACs showing the average percentage of Th2 cells (CD3+ CD4+ Gata3+) produced from Th0 splenocytes cultured under Th2 polarizing conditions (αCD3, αCD28, αIFNγ, IL2 and IL4) and exposed to vehicle, SR9243 or GW2965 treatment for 7 days. (**E**) FACs histogram showing the viability of polarized Th1 cells in response to vehicle, SR9243 or GW3965 with TCM (-upper panel) or RPMI1640 control media (-lower panel) for 24 h. (**F**) FACs data showing the mean percentage of Th1-polarized cells produced in response to DMSO, SR9243 or GW3965 treatment for 24 h with or without TCM cotreatment. (**G**) FACs data showing the number of viable Treg cells (CD4+ Foxp3+) produced from Th0 cells cultured under Treg differentiation conditions (αCD3, αCD28, αIFNγ, αIL12/αIL23, αIL4, IL6 and TGFβ) and treated with TCM or control media in tandem with DMSO, SR9243 or GW3965 for 7 days (**H**) FACs quantified viability of fully differentiated Tregs exposed to DMSO, SR9243 and GW3965 with or without TCM for 24 h. (**I**) FACs analysis showing the percentage of fully differentiated Tregs produced in response to DMSO, SR9243 or GW3965 with TCM or control media treatment for 24 h. For all experiments naïve CD4+ (Th0) splenocytes and lymphocytes were isolated from *C57BL6J* mice using a pan CD4+ negative selection affinity column (Miltenyi) and differentiated under Th1/Th2/Treg polarizing conditions as described. Cells were treated with 10 μM SR9243, 5 μM GW3965 or DMSO for 7 days or with LXR ligands and either 50% E0771 TCM or RPMI1640 control media for 24 h starting on day 6 of differentiation where stated. FACs histograms are representative results. All bar graphs are average percentages from three repeat experiments. *p < 0.05 1-Way ANOVA.

In addition to their effect on macrophage polarization, LXR agonists have been shown to inhibit CD4+ T-cell differentiation^37,38,40^. We decided to investigate whether tumor produced LXR ligands can directly influence the function, differentiation and polarization of CD4+ T-cells. Toward this goal, we treated CD4 + splenocytes, cultured under Th1/Th2 differentiation conditions, (as described previously^38^) with DMSO-vehicle, SR9243 or GW3965 in the presence of 50% E0771-TCM or control E0771 complete culture media for 7 days. These cells were then assessed for viability and markers of Th1 (CD3+ CD4+ Tbet+) or Th2 (CD3+ CD4+ Gata3+) polarization where relevant via FACs. Interestingly, SR9243 differentially enhanced Th1 differentiation as it robustly induced Tbet expression; a key marker of Th1 polarization (Fig. 2A,B). Notably, SR9243 significantly enhanced Th1 viability (Fig. 2C), yet had no effect on Th2 viability (Fig. 2D). As observed in previous reports^38^, LXR activation with GW3965 inhibited both Th1 and Th2 differentiation (Fig. 2C,D). Surprisingly, SR9243 induction of Th1 differentiation was not accompanied by enhanced IFNγ expression (Supplementary Fig. S2A,B). E0771-TCM also did not detectably modulate Th1 activity *in vitro* (Fig. 2E). However, SR9243 did effectively enhanced Th1 differentiation in the presence of TCM. In contrast GW3965 had strikingly inhibitory effects on Th1 viability in TCM-exposed cells (Fig. 2E,F). These results implied that E0771-LXR ligands did not directly modulate Th1 activity or differentiation, which aligned with our TNBC scRNA seq data. However, these results hinted that LXR inhibition with SR9243 may stimulate Th1 differentiation within the tumor microenvironment.

### SR9243 does not directly influence Treg differentiation and viability

Treg cells are an immunosuppressive cell type that is integrally involved in TNBC resistance to clinically used immunotherapies^49^. We decided to probe whether TCM and or synthetic LXR ligands can influence Treg differentiation and activity. We isolated CD4+ T-cells from *C57BL6J* mice and cultured them under Treg differentiation conditions with either vehicle, SR9243 or GW3965, in the presence of control media or 50% TCM for 7 days. In the absence of TCM, SR9243 reduced Treg viability whereas GW3965 had no significant effect (Fig. 2G,H). However, the inhibitory effect of SR9243 on Treg viability was lost upon TCM-exposure. In contrast, GW3965 selectively increased Treg viability and differentiation in TCM-exposed Tregs (Fig. 2G–I). Importantly, TCM alone did not influence Treg viability or differentiation, but interestingly LXR agonism improved Treg viability and induced FOXP3 expression, particularly in cells exposed to TCM (Fig. 2G–I). We also found that GW3965 treatment induced expression of the checkpoint blockade factor CTLA-4 in TCM-naïve Tregs (Supplementary Fig. S2C,D). These results suggested that TNBC-produced LXR ligands do not influence Treg activity but synthetic LXR agonists may promote immune tolerance in the context of the tumor microenvironment through Treg activation.

### LXR inhibition stimulates CD8+ T-cell activity

**Figure 3:**
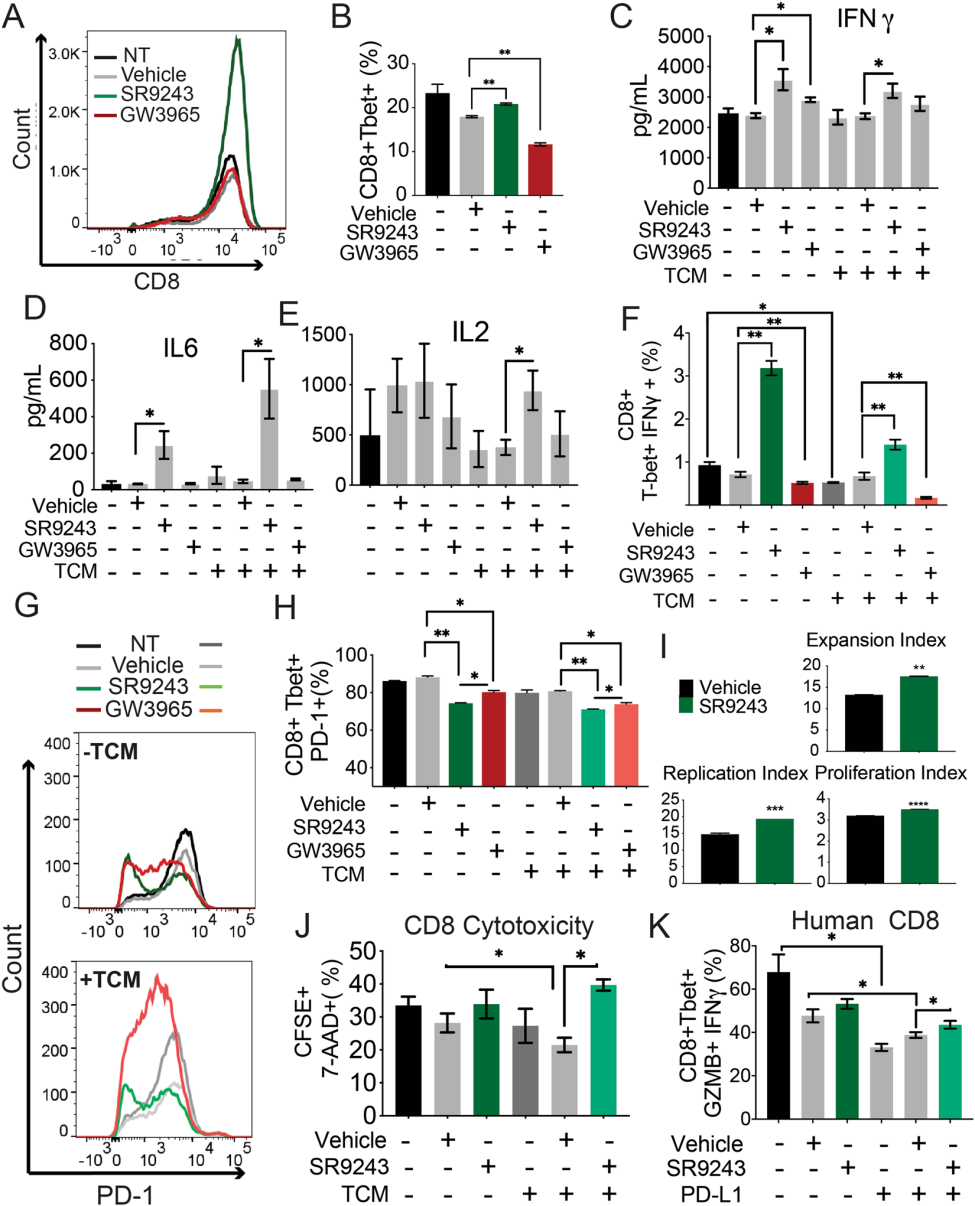
LXR inhibition activates CD8+ T-cells. (**A**) The number of CD8+ effector T-cells. (CD3+ CD8+ Tbet+) produced in response to 10 μM SR9243 or 5 μM GW3965 or DMSO vehicle treatment for 24 h as determined by FACs. (**B**) Percentage of CD8 + effector T-cells (CD3+ CD8+ Tbet+) produced from differentiated CD8+ T-cells treated with SR9243, GW3965 or vehicle for 24 h. Naïve CD8+ T-cells were isolated from C57BL6J mouse splenocytes using negative selection column purification (Militenyi) and cultured under CD8+ differentiation conditions (αCD3, αCD28, IL2 and IL7) for 4 days and exposed to LXR ligands for 24 h starting on day 3. (**C**–**E**) Cytometric bead assay showing CD8+ T-cell production of (C) IFNγ (**D**) IL6 and (**E**) IL2 in response to 10 μM SR9243 or 5 μM GW3965 for 24 h. Differentiated CD8+ T-cells were re-stimulated using 50 ng/mL/1 µg/mL PMA/Ionomycin for 4 h prior to cytokine quantification. (**F**) Average percentage of IFNγ expressing CD8+ effector T-cells (CD3+ CD8+ Tbet+) produced from differentiated CD8+ T-cells exposed to vehicle, 10 μM SR9243 or 5 μM GW3965 in the presence or absence of TCM. Cells were treated with LXR ligands for 24 h prior to FACs analysis **(G**) FACs histogram showing PD-1 expression in CD8+ effector T-cells treated with vehicle, SR9243 or GW3965 in the presence of 50% E0771 TCM or relevant control media (RPMI1640) for 24 h (**H**) Average PD-1 expression in CD8+ effector T-cells treated with vehicle, SR9243 or GW3965 for 24 h as determined by FACs analysis. (**I**) Carboxyfluorescein succimidyl (CFSE) proliferation assay of differentiating CD8+ T-cells treated with SR9243 or DMSO. Column isolated CD8 T cells were labeled with 1 µM CellTrace^TM^ CFSE (ThermoFisher) for 20 minutes at room temperature, then subjected to CD8+ T-cell differentiation conditions for 96 h, as described, in the presence of vehicle or 10 µM SR9243. Expansion, proliferation and replication indices were then determined via FACs analysis of CFSE dilution profiles using Flow-Jo. (**J**) Cell killing/CD8+ T-cell cytotoxicity assay showing CD8+ T-cell destruction of cultured E0771 cells in response to SR9243, with or without TCM co-treatment. CD8+ T-cells were differentiated for 4 days then exposed to 10 μM SR9243 along with 50% E0771 TCM or RPMI1640 for 24 h. CD8+ cells were exposed to CD3/CD28 activation in the presence of target cells; E0771 cells were pre-stained with 1 µM CFSE and 4 µg/mL 7-Aminoactinomycin-D (7-AAD). The percentage of E0771 cells (CFSE + 7-AAD+) were then quantified via FACs. (**K**) Mean percentage of IFNγ + GZMB+ human CD8+ T-cells (CD3+ CD8+ Tbet+) produced in response to SR9243, with or without PD-L1 immune-checkpoint suppression. CD8+ T-cells were isolated from PMBCs from healthy female volunteers via negative selection using the RosetteSep™ Human CD8+ T Cell Enrichment Cocktail. Cells were then exposed to 5 μg/mL purified human PD-L1 protein or vehicle (PBS) in the presence of 100 nM SR9243 for 24 h. *p < 0.05 as determined by 1-Way ANOVA.

CD8+ effector T-cells (CD3+ CD4- CD8+ Tbet+) and effector memory T-cells (CD3+ CD4- CD8+ Tbet+ Eomes+) are essential to tumor cell killing and immunological memory, respectively. However, although LXR ligand activity has been previously studied in CD4+ T-cells, the direct effect of LXR modulators on CD8+ T-cell activity has not been described. Therefore, we assessed whether SR9243 or GW3965 could influence CD8+ T-cell differentiation and activation. First, we treated fully differentiated CD8+ T-cells with GW3965 or SR9243 for 24 h followed by stimulation with PMA/Ionomycin for 4 h, then subjected them to FACs analysis with cognate CD8+ T-cell phenotyping markers. SR9243 stimulated CD8 + effector T-cell differentiation as it increased the percentage of cells expressing the two key CD8 effector T-cell markers CD8 and Tbet (Fig. 3A,B). Similarly, SR9243 increased the viability of effector memory cells (Supplementary Fig. S3A,B) but had no effect on memory cell differentiation, as the percentage CD8+ Tbet + Eomes + triple positive T-cells were unchanged (Supplementary Fig. S3C). SR9243 boosted *Ifnγ* mRNA expression (Supplementary Fig. S3D) and IFNγ and IL6 protein production in CD8 + effector T-cells (Fig. 3C,D). Compared to SR9243, GW3965 also modestly induced *Ifnγ* mRNA expression (Supplementary Fig. S3D), but had no effect on IFNγ protein synthesis or release (and Supplementary Fig. S3D, S3E). IL-2 production, which positively correlates with TNBC relapse and survival, and is a clinical target for the treatment of metastatic TNBC^50^, was intriguingly only significantly induced in TCM-exposed CD8 + effector T-cells treated with SR9243, but not GW3965 (Fig. 3E). SR9243 also stimulated an increase in the percentage of IFNγ positive cells (Fig. 3F) suggesting an overall clonal shift in CD8 T-cell activity in addition to changes in protein production. Notably, TCM reduced the percentage of IFNγ-expressing CD8 + effector cells (Fig. 3F), but did not significantly reduce that of effector memory CD8+ T-cells (Supplementary Fig. S3E). SR9243 also inhibited PD-1 expression, a receptor for PD-L1, in effector and effector memory T-cells (Fig. 3G,H and Supplementary Fig. S3F,G). Interestingly, like SR9243, GW3965 similarly modulated PD-1 expression on effector and effector memory CD8 cells (Fig. 3G,H and Supplementary Fig. S3F and G). These observations indicated that LXR inhibition with SR9243 may directly stimulate CD8+ T-cell activity and that LXR ligands may influence PD-1 expression.

### SR9243 stimulates CD8+ T-cell clonal expansion

As SR9243 showed an ability to stimulate CD8 + inflammatory activity we decided to explore if LXR blockade can stimulate CD8+ T-cell clonal expansion, which is vital to mounting an effective anti-tumor immune response. To measure proliferation CD8+ T-cells were stained with carboxyfluorescein succimidyl (CFSE) then differentiated for 96 h in the presence of SR9243 or vehicle control, exposed to TCM and CD3/CD28 stimulation and then analyzed for CFSE staining via FACs. Remarkably, SR9243 had a stimulatory effect on key aspects of CD8+ T-cell clonal expansion (Fig. 3I) as it specifically enhanced the Proliferation Index, Expansion Index, Replication Index (Fig. 3I) and Division Index (Supplementary Fig. S3H) compared to vehicle. However, SR9243 did not increase the Dilution Index suggesting that it did not increase the proliferation rate of the original mitotic pool of CD8+ T-cells (Supplementary Fig. S3H). These observations highlight that SR9243 can effectively stimulate CD8+ T-cell clonal expansion in response to inflammatory stimulation.

### LXR inhibition stimulates CD8 + cytotoxicity

Considering the effect of SR9243 on CD8+ proliferation and inflammatory activity, we decided to determine if SR9243 could promote CD8+ effector T-cell cytotoxicity. Toward this goal, we differentiated CD8+ T-cells and exposed them to 100 nM SR9243, 100 nM GW3965, DMSO and or TCM for 24 h then performed a CD8 T-cell cytotoxicity assay. CD8 T-cells were co-cultured with intact E0771 (1:1 ratio) cells that were previously stained with CFSE for 15 minutes. Following exposure to CD8+ T-cells, E0771 cells were stained with the viability dye 7-aminoactinomycin D (7-AAD). E0771 cell death; a direct indication of CD8 T-cell toxicity was then confirmed using FACs quantification of the percentage of dead E0771 cells or CFSE+ 7-AAD+ cells. As predicted TCM reduced CD8+ T-cell cytotoxicity (Fig. 3J). SR9243, conversely rescued TCM-mediated suppression of CD8 T-cell cytotoxicity (Fig. 3J) in TCM-exposed cells, but had no effect on CD8 T-cell activity in TCM naïve cells. In contrast, GW3965 failed to stimulate CD8+ TCD8+ T-cell cytotoxicity and in fact displayed a mild inhibitory effect (Supplementary Fig. S3I). These results suggest that tumor-produced LXR ligands can repress CD8+ cytotoxic activity and lastly that LXR inhibition via SR9243 could be used to promote CD8+ T-cell mediated tumor cell destruction.

### LXR inhibition activates human CD8+ T-cells

Bearing in mind the evidence generated thus far suggesting that LXR ligands may influence mouse CD8+ T-cell activity, we decided to assess if the stimulatory activity of SR9243 is conserved in human CD8+ T-cells. Circulating human CD8+ T-cells were therefore isolated from the blood of healthy female donors and cultured with recombinant human PD-L1 protein or vehicle control, to simulate PD-1-mediated CD8 T-cell suppression. We then measured the effect of SR9243 or on CD8+ T-cell activation using FACs analysis. Interestingly, SR9243 selectively induced IFNγ and GZMB expression only in CD8+ effector T-cells exposed to PD-L1 (Fig. 3K). In accord with this, GW3965 had no effect on control treated human CD8+ T-cells, yet additively repressed IFNγ expression when combined with PD-L1 (Supplementary Fig. S3J). These results indicated that SR9243 may be used to effectively stimulate CD8+ T-cell activity in humans and highlight that LXR may similarly regulate CD8+ T-cell activity in humans and mice.

### SR9243 enhances CD8+ T-cell mitochondrial activity

**Figure 4:**
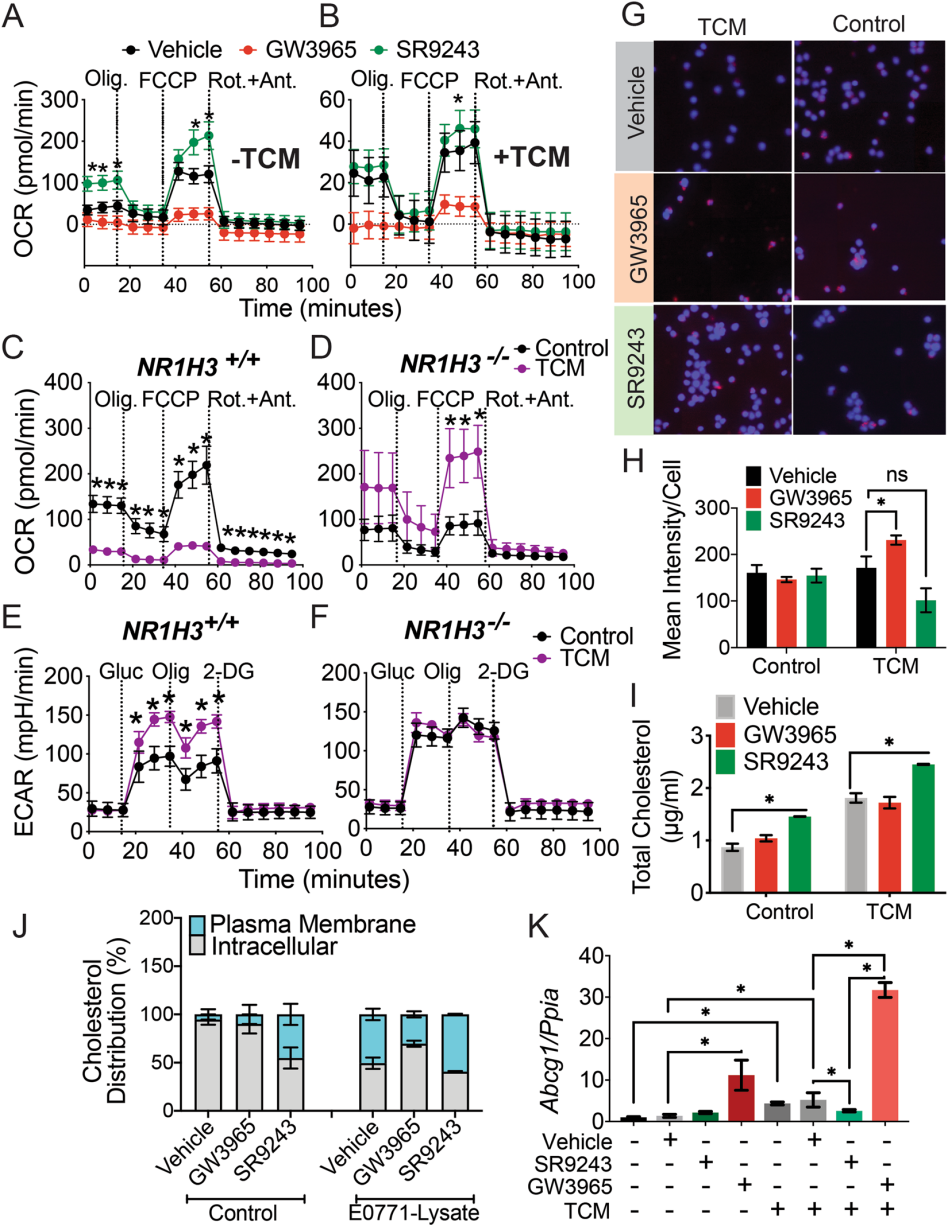
LXR inhibition enhances CD8+ T-cell mitochondrial metabolism. (**A**) Extracellular flux analysis (Seahorse) showing the oxygen consumption rate (OCR) of mouse CD8+ T-cells exposed to 50% RPMI1640 control culture media (−TCM) and either DMSO (vehicle), SR9243 or GW3965 for 4 h. (**B**) OCR of mouse CD8+ T-cells cultured in 50% E0771-TCM (+TCM) and treated with vehicle, SR9243 or GW3965 for 24 h. (**C**,**D**), Mitochondrial profiling (OCR) of mature CD8+ T-cells isolated from wildtype *C57BL6J* (*Nr1h3*^+/+^) or *LXRα* knockout mice (*Nr1h3*^−/−^) mice cultured in 50% TCM (TCM) or relevant control media (Control) for 24 h prior to analysis. (**E,F**), Glycolytic metabolism profiling showing the extracellular acidification rate (ECAR) of mature *Nr1h3*^+/+^ or *Nr1h3*^−/−^ CD8+ T-cells cultured in TCM (TCM) or control media (Control) for 24 h. OCR and ECAR were quantified simultaneously using the XF96 Seahorse bioanalyzer (Agilent). CD8+ T-cells were column isolated from mouse splenocytes and differentiated for 3 days as described and treated with DMSO 100 nM SR9243 or 100 nM GW3965 for 4 h then plated in Seahorse base-media prior to analysis. Glycolytic and mitochondrial profiles were determined using the XF-Mitochondrial stress-test or the Glycolysis stress-test assay (Agilent) as per manufacturer’s instructions. Olig.: Olygomycin, FCCP: carbonylcyanide-4 (trifluoromethoxy) phenylhydrazone Rot + Ant: Rotenone and Antimycin A, Gluc: glucose, 2-DG: 2-deoxyglucose. (**G**) Oil-Red-O neutral lipid staining showing lipid content of wildtype CD8+ T-cells exposed to 100 nM SR9243 or 100 nM GW3965 for 24 h. Micrographs shown are representative images from 2 repeat experiments. (**H**) Mean lipid content quantified from images of Oil-Red-O stained CD8+ T-cells. Oil-Red-O and DAPI nuclear staining quantified using ImageJ. (**I**) Total cholesterol content of CD8+ T-cells treated with 100 nM SR9243 and 100 nM GW3965 for 24 h. (**J**) Ratio of membrane-versus-intracellular cholesterol in mature CD8+ T-cells treated with vehicle or 100 nM SR9243 or 100 nM GW3965 for 24 h. Cholesterol content measured using Amplex-Red Assay (Sigma) using manufacturer’s instructions. (**K**) RT-QPCR-quantified expression *Abcg1* in CD8+ T-cells in response to LXR ligands and TCM. For staining and RT-QPCR CD8+ T-cells were column isolated and differentiated as described, 3 days then exposed to the LXR ligands for 24 h. *p < 0.05 determined by 2-Way ANOVA or 1-Way ANOVA where relevant.

We decided to identify the mechanisms through which LXR may be triggering the observed effects on CD8+ effector T-cell activity. We theorized that LXR may augment CD8+ metabolism in response to tumor-associated antigens. To investigate this, we exposed fully-differentiated CD8+ T-cells to 100 nM SR9243 or GW3965 for 24 h then stimulated them with PMA/Ionomycin followed by exposure to E0771-TCM and whole cell lysate for 4 h. CD8+ effector T-cells (2 × 10^5^ per well) subjected to these conditions were then isolated and plated in Seahorse media and subjected to mitochondrial profiling using the Seahorse XF96e Extracellular Flux Bioanalyzer (Agilent). Remarkably, TCM exposure depressed mitochondrial activity as the maximum oxygen consumption rate (OCR) declined significantly from 150 in tumor naïve cells to 40 pmol/min in tumor exposed cells (Fig. 4A,B). Notably, 100 nM of SR2943 alone (−TCM) substantially increased the mitochondrial activity of CD8+ T-cells (Fig. 4A) as it stimulated an increase in basal and maximal respiration, without compromising mitochondrial spare respiratory capacity (Fig. 4A and Supplementary Fig. S4A). In contrast, cells treated with 100 nM GW3965 alone (−TCM) displayed almost completely blunted basal and maximal mitochondrial respiration (Fig. 4A and Supplementary Fig. S4A).

In addition to the observed effects on mitochondrial respiration, TCM and tumor-antigen exposure significantly suppressed the maximum glycolytic capacity of CD8 T-cells as the extracellular acidification rate (ECAR) was reduced in tumor-exposed, compared to naïve cells (Supplementary Fig. S4B,C). Surprisingly, neither SR9243 nor GW3965 influenced CD8 T-cell glycolytic output (Supplementary Fig. S4B–D). To gain clearer insight into the role of LXR in mitochondrial regulation we further assessed whether SR9243 could be augmenting CD8+ T-cell L-lactate production; a direct readout of glycolytic flux. Biochemical analysis indicated that L-lactate content in SR9243-treated CD8 T-cells was also not significantly altered by SR9243 (Supplementary Fig. S4E), although there was a strong trend (p = 0.508) toward elevated L-lactate production (Supplementary Fig. S4E). These observations highlight that SR9243 can effectively stimulate CD8+ T-cell mitochondrial respiration and suggests that LXR activation within TNBC tumors may have an inhibitory effect on CD8 + mitochondrial metabolism.

### Loss of LXRα enhances the mitochondrial activity of CD8+ T-cells

Pharmacological modulation of LXR using SR9243 and GW3965 revealed a possible suppressive role of LXRs in CD8+ T-cell mitochondrial respiration. To exclude off-target drug effects as contributary factors driving our observations, we exposed fully differentiated CD8+ T-cells from female *Lxrα* knockout mice (*Nr1h3*^−/−^) and wildtype littermate controls (*Nr1h3*^+/+^) to TCM and tumor antigens and profiled their mitochondrial activity and glycolytic output as before. In accord with our prior results, TCM reduced CD8+ T-cell mitochondrial activity in wildtype cells (Fig. 4C). In contrast, *Lxrα* knockout CD8+ T-cells cells displayed a robust elevation of mitochondrial activity in response to TCM (Fig. 4D). Intriguingly, *Lxrα* knockout CD8+ TCD8+ T-cells robustly elevated their maximal and spare respiratory capacity in response to tumor antigens (Supplementary Fig. S4I). Conversely, maximal, basal and spare respiratory capacity were suppressed in TCM-exposed wildtype cells (Supplementary Fig. S4I). *Lxrα* deletion also enhanced the basal glycolytic rate of CD8+ T-cells relative to wildtype (Fig. 4E,F and Supplementary Fig. S4J). However, *Lxrα* null CD8+ T-cells did not increase their glycolytic output in response to TCM as observed in wildtype CD8+ cells (Fig. 4E,F). Strangely, whereas wildtype cells showed increased glycolytic capacity and reduced basal glycolytic rate in response to TCM, *Lxrα* null CD8+ T-cells were able to increase their glycolytic reserve despite having an already elevated basal glycolytic output (Supplementary Fig. S4J). All these results collectively suggested that LXR to restrains CD8+ T-cell mitochondrial glycolytic metabolism and plasticity and also implied that TNBC tumors may avoid immune-destruction by inducing LXR and stunting T-cell metabolism.

### Lipid synthesis in CD8+ T-cells is not influenced by TNBC-LXR ligand production

In addition to an oxidative and glycolytic metabolic serge, effective CD8+ T-cell activation relies on robust induction of lipid synthesis, which uniquely provides a source of energy and molecular building blocks for membrane expansion^6,51^. First, to quantify the effect of LXR modulation on lipid metabolism, we assayed the neutral lipid content of CD8+ T-cells exposed to E0771-TCM in response to SR9243, GW3965 or vehicle using Oil-Red-O staining. Interestingly, we found that neutral lipid content was not modulated by TCM or SR9243, but was significantly increased by GW3965 in TCM-exposed CD8+ T-cells. (Fig. 4G,H). These results suggest that TNBC LXR ligands do not significantly influence lipogenesis or lipid uptake in CD8+ T-cells, but also highlight that LXR activation, particularly by synthetic agonists, can upregulate CD8+ T-cell lipogenesis.

### SR9243 promotes plasma membrane localization of cholesterol in CD8+ T-cells

Another central event in CD8 + activation is the localization of cholesterol to the plasma membrane which facilitates immune-synapse formation and is vital for tumor cell destruction^52^. LXR has been shown to have a role in regulating cholesterol metabolism in CD8+ T-cells, and cholesterol subcellular localization and membrane composition in numerous cell types^53,54^. Therefore, we assessed the total cholesterol content of tumor-naïve or TCM exposed CD8+ T-cells treated with DMSO-vehicle, SR9243 or GW3965 for 24 h using an AmplexRed cholesterol assay kit (ThermoFisher). Total cholesterol in CD8+ T-cells were increased by exposure to TCM overall with GW3965 interestingly having no effect on total cholesterol content (Fig. 4I). Conversely, SR9243 enhanced cholesterol content in both naïve and tumor exposed CD8+ T-cells (Fig. 4I). Importantly, in addition to increasing cholesterol production overall, in tumor exposed CD8+ T-cells, SR9243 significantly enhanced the enrichment of cholesterol in the plasma membrane compared to vehicle and GW3965 treated cells (Fig. 4J and Supplementary Fig. S4F,G). Interestingly, SR9243 did not influence cholesterol content in CD4+ Th1 or Th2 cells, while GW3965 induced a mild but significant increase in total cholesterol in Th2 cells only (Supplementary Fig. S4H). This observation suggested that cholesterol localization and or reverse transport may be uniquely regulated in CD8+ T-cells. In order to identify what was driving increased plasma membrane cholesterol accumulation in SR9243 treated CD8+ cells we quantified the expression of LXR and the LXR-regulated ABC-transporters *Abca1* and *Abcg1* in CD8+ T-cells using QPCR. In CD8+ T-cells *Lxrα* and *Lxrβ* expression was increased by exposure to TCM in tandem with *Abcg1*, known to be the main cholesterol transporter in CD8+ T-cells^55^ (Supplementary Fig. S4K,L and Fig. 4K). Mostly importantly, *Abcg1* expression in response to TCM was completely blocked by SR9243, yet substantially induced by GW3965 (Fig. 4K). Contrary to this, *Abca1* a cholesterol transporter also regulated by LXR, but lacking a prominent role in cholesterol efflux in T-cells^55^ was not induced by exposure to TCM (Supplementary Fig. S4M). However, *Abca1* was also similarly repressed by SR9243 and extensively activated by GW3965 (Supplementary Fig. S4M). These observations suggest that SR9243 stimulated an increase in cholesterol content in CD8+ T-cells and facilitated cholesterol localization to the plasma membrane possibly via inhibition of *Abcg1* expression. Our observations also indicate that *Abcg1* may be selectively induced in CD8+ T-cells as well as macrophages within the TNBC tumor microenvironment.

### LXR Inhibition induces immune-dependent tumor destruction *in vivo*

**Figure 5:**
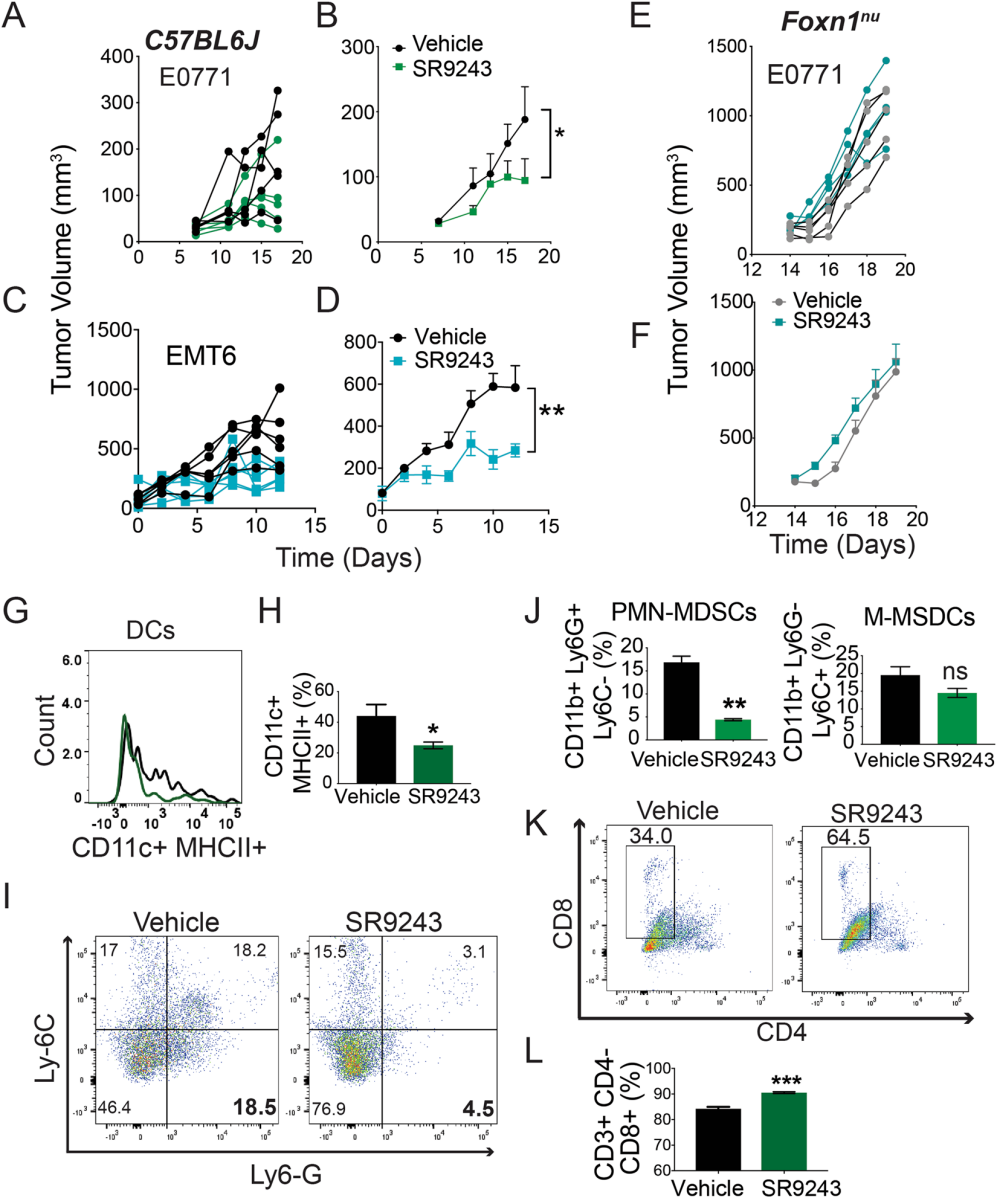
SR9243 induces immune-mediated TNBC tumor destruction *in vivo*. (**A**,**B)** E0771-tumor volumes in *C57BL6J* mice treated with vehicle (DMSO:Tween80:PBS/10:10:80) or 60 mg/kg SR9243 for 16 days. A: Line graph showing growth of individual tumors. B: Line graph showing the mean tumor volume for each group. (**C**,**D)** EMT6-tumor volumes in *C57BL6J* mice treated with vehicle or 60 mg/kg SR9243. C: Line graph showing growth of individual tumors. (**D**) Line graph showing the mean tumor volume for each group. (**E**,**F)** E0771-tumor volumes in *Foxn1*^*nu*^ nude mice treated with vehicle or 60 mg/kg SR9243. C: Line graph showing growth of individual tumors. (**D**) Line graph showing the mean tumor volume for each group. Mice were implanted with 1 × 10^6^ E0771 or 2 × 10^5^ EMT6 cells in the lower left mammary fat pad. Tumors were allowed to establish to 50 mm^3^ then mice were treated with 60 mg/kg SR9243 or vehicle (10:10:80-DMSO:Tween80:PBS) once daily for 16 days (n = 6). Tumor volume was calculated using perpendicular caliper measurements (1/2LW^2^). *p < 0.05 as determined by 2-Way-ANOVA (**G**) Histogram displaying the number of tumor resident dendritic cells (CD11C + MHCII+) in SR9243 versus vehicle treated E0771 tumor-bearing *C57BL6J* mice quantified by FACs. (**H**) Average percentage of tumor resident DCs (CD11c + MHCII+) in tumors (**I**) FACs dot-plot showing the percentages of tumor resident myeloid derived suppressor cells (MDSCs: PMN-MDSCs: CD11B + Ly6G + Ly6C−) and monocytic MDSCs (M-MDSCs: CD11b + Ly6G-Ly6C+) resident in E0771-tumors implanted in *C57BL6J* mice. (**J**) Average percentage of tumor resident PMN-MDSCs and M-MDSCs (right panel) in control versus SR9243 groups determined by FACs (n = 6). (**K**) FACs-dot plot showing the percentage of tumor-resident CD8+ T-cells in response to SR9243 treatment. (**L**) Average percentage of CD8+ T-cells among tumor infiltrating lymphocytes in vehicle and SR9243 treated tumors.

With our combined observations *in-vitro* we focused on testing whether SR9243 could stimulate a potent anti-tumor immune response *in vivo*. To generate an *in vivo* model of TNBC we orthotopically implanted *C57BL6J* mice with syngeneic E0771 (1 × 10^6^) or EMT6 (2 × 10^5^) cells. Mice were dosed via *i*.*p*. with 60 mg/kg of SR9243, a maximally efficacious dose as previously determined^14^, or vehicle (10:10:80-DMSO:Tween80:PBS). Wild-type mice with E0771 or EMT6 orthotopic allograft tumors displayed reduced tumor growth when dosed with SR9243 (Fig. 5A–D). We were able to determine that E0771 are resistant to the direct cytotoxic effects of SR9243 (Supplementary Fig. S5A). Interestingly, E0771 allograft tumors like clinical TNBC are known to be resistant to PD-1 checkpoint blockade inhibitors. Therefore, in order to determine if the anti-tumor effect of SR9243 was immune-dependent, we orthotopically inoculated immune-compromised *Foxn1*^*nu*^ mice with E0771 cells (1 × 10^6^) and similarly dosed them with SR9243. Interestingly, in *Foxn1*^*nu*^ mice, E0771-tumor volume was unaffected by SR9243, in contrast to that of wildtype mice (Fig. 5E,F). This outcome suggested that SR9243 was most likely disrupting tumor growth through stimulation of immune mediated tumor clearance. Importantly, SR9243 produced an anti-tumor immune response without overt toxic effects, as total body weight was not affected by SR9243 administration, as seen in previous studies^14^ (Supplementary Fig. S5B–D). These results emphasized that SR9243 may have utility as a small molecule immunotherapeutic.

### SR9243 promotes DC migration and suppresses MDSC and Treg populations

To characterize the immunotherapeutic activity of SR9243, we FACs-profiled tumor-infiltrating and lymph node resident DCs, myeloid-derived suppressor cells (MDSCs), CD8+ T-cells and Tregs from tumor-bearing *C57BL6J* mice treated with SR9243. SR9243-treated mice exhibited a potent reduction in DCs within the tumor microenvironment with a significant concomitant increase in lymph-node-resident DCs relative to vehicle control (Fig. 5G,H and Supplementary Fig. S5E,F). Surprisingly, SR9243 also reduced the population of tumor-resident polymorphonuclear MDSCs (PMN-MDSCs: CD11C + Ly6G + Ly6C-) (Fig. 5I,J), a central immune suppressive cell type known to underlie poor clinical outcomes and resistance to immunotherapy^9,56,57^. Conversely, monocytic MDSCs (CD11C + Ly6G-Ly6C+), not known to be regulate tumor-immune interactions, were not significantly affected by SR9243 (Fig. 5I,J). Lastly, intra-tumor infiltration of Tregs (CD3 + CD4 + FOXP3) was also disrupted by SR9243 (Supplementary Fig. S6A). These findings assert that SR9243 facilitated DC lymph node migration and suppressed MDSC and Treg tumor infiltration *in vivo*.

### SR9243 stimulates CD8+ T-cell tumor infiltration and anti-tumor activity *in vivo*

**Figure 6:**
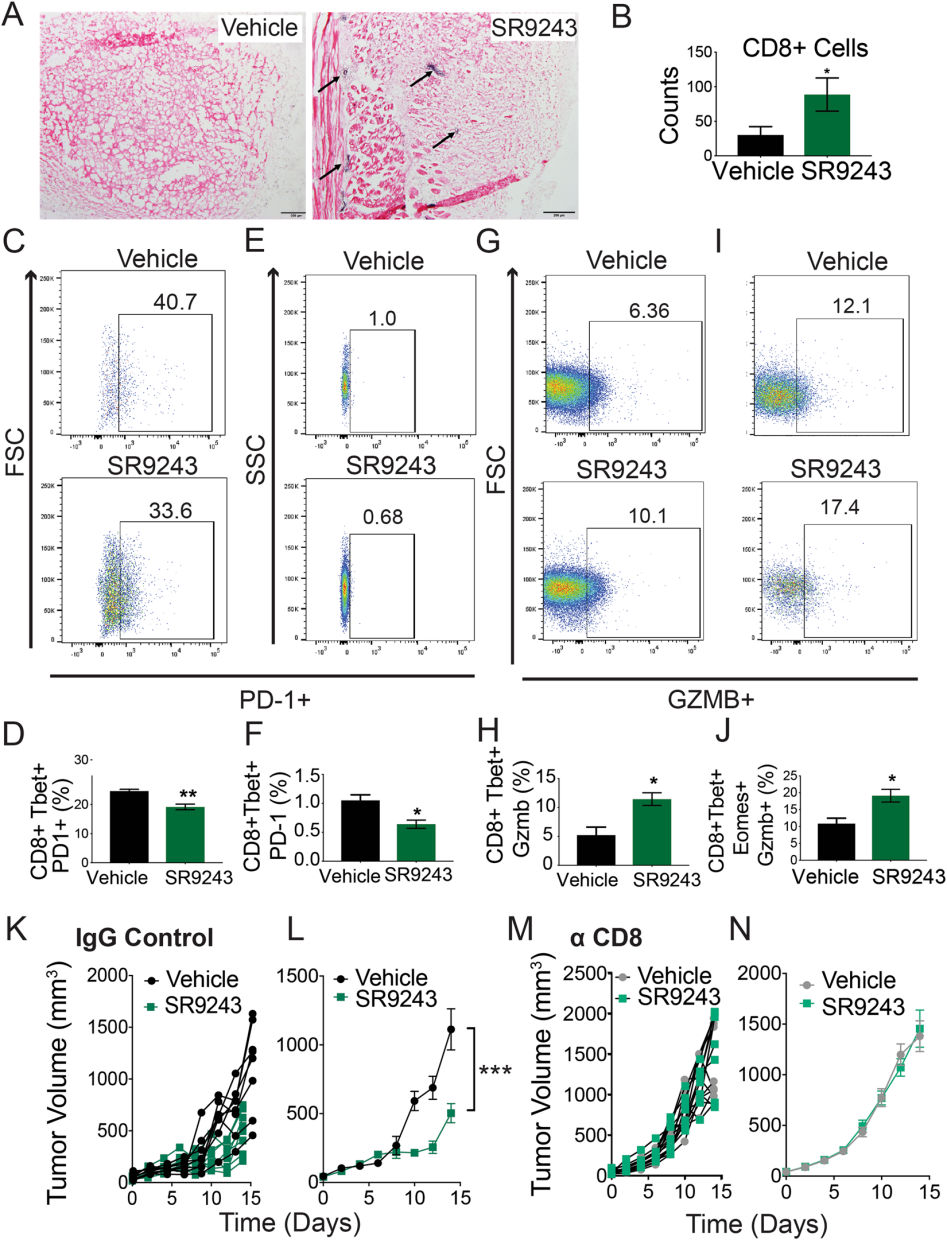
SR9243 immunotherapeutic activity is CD8+ T-cell dependent. (**A**) Immunohistochemical staining showing CD8+ infiltration into E0771 tumors from SR9243 or vehicle control treated mice. (**B**) Average number of CD8+ T-cells quantified in vehicle versus SR9243 treated tumors (n = 6). The number of CD8+ cells were quantified using ImageJ. *p < 0.05 determined by student’s t-test. (**C**) FACs dot-plot showing the percentage of tumor resident CD8+ effector T-cells expressing PD-1 in vehicle and SR9243 treated mice (**D**) Average percentage of tumor-resident CD8+ effector T-cells expressing PD-1+ in vehicle versus SR9243 treated groups (n = 6). (**E**) FACs dot-plot showing the percentage of lymph-node resident CD8+ effector T-cells that are positive for PD-1 expression in vehicle and SR9243 treated mice (**F**) Average percentage of lymph-node resident CD8+ effector T-cells expressing PD-1+ in vehicle versus SR9243 treated groups (n = 6). (**G**) FACs dot-plot showing the percentage of lymph node resident CD8+ effector memory T-cells expressing GZMB in control or SR9243 treated mice. (**H**) Average percentage of GZMB expressing effector T-cells in the draining lymph-nodes of vehicle and SR9243 treated mice. (**I**) FACs dot-plot showing the percentage of lymph node resident CD8+ T-cells expressing GZMB in vehicle and SR9243 mice. (**J**) Mean percentage of lymph-node resident CD8+ T-cells expressing GZMB in vehicle and SR9243 treated mice (**K**) Individual E0771 tumor allograft volumes for individual mice receiving Rat IgG2A and dosed with vehicle or 60 mg/kg SR9243. (**L**) Mean tumor allograft volume for vehicle and SR9243 treated groups in J. (**M**) Individual E0771-allograft volumes for SR9243 and vehicle treated mice (**N**) Mean E0771-tumor allograft volumes for mice depleted of CD8 T-cells and treated with vehicle or SR9243 in K. For CD8+ T-cell depletion tumor bearing mice were dosed with αCD8 antibody (Bio-X-Cell) (or Rat IgG control) every three days for 9 days. Mice were then treated with 60 mg/kg SR9243 or vehicle once daily for 14 days (n = 10). *p < 0.05 ***p < 0.001 as determined by student’s t-test of 2-Way ANOVA.

Further analysis revealed that intratumor infiltration by CD8 + effector T-cells (CD3 + CD8+ Tbet+) was also pointedly increased by SR9243 when quantified using FACs (Fig. 5K,L) or immunohistochemistry (IHC) (Fig. 6A,B). CD8+ T-cell populations were similarly elevated in tumor draining lymph nodes of SR9243-treated E0771 and EMT6-tumor bearing mice (Fig. 6A,B and Supplementary Fig. S6B–D). In agreement with our observations *in vitro*, PD-1 expression in tumor resident CD8+ T-cells was inhibited by SR9243 (Fig. 6C,D). Similarly, lymph-node resident CD8 + effector cells displayed a reduction in PD-1 expression (Fig. 6E,F). Expression of the serine protease GZM-B, in lymph node resident effector (Fig. 6G,H) and effector memory (Fig. 6I,J) CD8+ T-cells was also effectively induced by SR9243. These results indicated that SR9243 can stimulate immune-mediated tumor destruction by inducing CD8+ T-cell activity *in vivo*.

### SR9243 immunotherapeutic activity is CD8+ T-cell dependent

Considering the ability of SR9243 to stimulate CD8+ T-cell activity *in vivo*, we tried to determine whether SR9243 immunotherapeutic activity was CD8+ T-cell dependent. We therefore orthotopically inoculated wildtype *C57BL6J* mice with E0771 cells then depleted them of CD8+ T-cells, using a CD8 + depleting Antibody (BioXcell). Tumor bearing mice were then dosed with 60 mg/kg SR9243 or vehicle for 16 days. As expected E0771 tumor growth in IgG-control treated mice was significantly reduced in in response to SR9243 (Fig. 6K,L and Supplementary Fig. S6E,G). However, tumors in CD8 + depleted mice, did not respond to SR9243 treatment (Fig. 6M,N and Supplementary Fig. S6E–G). These results affirmed that SR9243 stimulated immune-mediated tumor destruction in a CD8+ T-cell dependent manner.

## Discussion

Even with the recent approval of the first FDA-approved immunotherapy drug Atezolizumab, which is limited to the treatment of PD-L1 positive tumors, TNBC presents a clinical challenge. It is therefore imperative that novel mechanisms of TNBC immunotherapy resistance are identified and exploited in order to engineer novel treatments. Here we show that the Liver-X-Receptor is a putative drug-target for TNBC immunotherapies. Using sc-RNA-seq data from diverse breast cancer tumor subtypes we establish that TNBC tumors have uniquely elevated activation of LXR signaling, specifically in tumor-resident myeloid cells. Moreover, we demonstrate that TNBC tumor cells produce endogenous LXR ligands that activate LXR signaling in macrophages as well cytotoxic CD8 T-cells. In accord with this we also showed that TNBC-produced LXR ligands can inhibit macrophage M1 polarization. We also illustrate for the first time that LXR can inhibit CD8+ T-cell differentiation, activation, cytokine production, cholesterol mobilization, clonal expansion and cytotoxicity. Furthermore, we provide evidence that suggests that LXR activation inimitably suppresses immune function through disruption of CD8+ T-cell metabolism. Lastly, we confirmed that LXR inverse agonists can be utilized to effectively stimulate TNBC tumor destruction *in vivo*.

We have seen in previous studies that SR9243 can promote oxidative phosphorylation and downregulate the elevated glycolytic output or Warburg metabolism in tumor cells or premalignant cells. Importantly too, SR9243 is not known to influence glucose metabolism in normal cells^14,41,42,58–60^. Activated CD8+ T-cells have an energetic profile and metabolic requirements that closely mirror that of proliferating malignant cells. However, the response of CD8 T-cell to LXRα loss, we have seen here, is dissimilar to that observed in cancer cells or normal cells in response to pharmacological LXR inhibition. SR9243 stimulated mitochondrial metabolism without downregulating glycolytic activity in CD8+ T-cells. Yet genetic LXRα ablation strongly elevated basal glycolytic activity as well as enhanced mitochondrial function of CD8 T-cells. These observations can possibly due to inherent differences in a genetic versus and a drug-mediated approach, but point to a unique role of LXR in CD8 + metabolic function. For CD8 T-cells heightened oxidative phosphorylation and glycolytic flux are inherently coupled to inflammatory signaling. Prior studies have defined diverse mechanisms of LXR-mediated immune suppression in melanoma and hematological cancers^8,17,20^. Our findings further hint that LXR’s ability to constrain glucose metabolism may be an unexplored mechanism through which LXR mediates its potent anti-inflammatory activity in lymphoid and myeloid cells. Whether disruption of mitochondrial function in immune cells is crucial to LXR-mediated immune suppression in other types of solid malignancies is uncertain.

Using patient-derived samples we have shown that LXR pathway activation is a unique feature of tumor-infiltrating myeloid cells in TNBC. Using *in vitro* models that utilized TCM from human and mouse breast cancer cell lines we confirmed that TNBC-produced LXR ligands promoted tolerogenic (M2) macrophage polarization. However, in CD4 T-cells, while synthetic LXR ligands display consistent modulation of immune function, tumor-produced LXR ligands had weak or mixed results. Considering LXR ligands are a diverse group of cholesterol metabolites with distinct binding affinities, contrasting stability abundance, and are able to differentially regulate LXR activity, this finding is not overly surprising. Some of the limited responses obtained may be due to the relatively low abundance or instability of endogenous LXR ligands in culture. Contrasting responses to tumor LXR-ligands between T-cells and macrophages may also be due to inherent differences in cholesterol metabolism or LXR-pathway regulation between these immune cell types. Importantly in this study we did not identify what specific endogenous LXR ligands are produced by TNBC tumors. Elucidating what specific tumor produced ligands may mediate immune suppression in TNBC is the subject of ongoing investigation.

In this study SR9243 unexpectedly reduced PMN-MDSC populations, a clinically relevant immune-suppressive cell-type. Similar effects have been observed for the LXR ligand RGX-104, currently in Phase 1 dose escalation clinical trials. However, considering RGX-104 and SR9243 modulate LXR activity in different ways, the mechanism through which SR9243 inhibits MDSCs is as yet unclear. However, the discoveries reported here collectively suggest that the development of future TNBC immunotherapies should include LXR inverse-agonists.

## Materials and Methods

### Animals

All animal studies were conducted with approval by the Saint Louis University Institutional Animal Care Committee (IACUC; protocol #2368) and all experiments were carried out with strict accordance with relevant guidelines and regulations. Six-week-old C57BL/6J mice were obtained from Jackson Laboratories. Athymic nude [Crl:NU(NCr)-Foxn1nu] mice were obtained from Charles River Laboratories. Mice were housed in sterile ventilated cages under a standard dark light cycle. They were provided a standard chow diet and water *ad libitum*.

### Orthotopic allograft model

E0771 or EMT6 cells were harvested using trypsin/EDTA (Caisson), washed with PBS, and resuspended in HBSS containing Matrigel (Corning). E0771 (2 × 10^6^ or 3 × 10^6^) or EMT6 cells (2 × 10^6^) were planted subcutaneously into the mammary fat pad of 6-week-old C57BL/6J mice (Jackson Laboratories) or athymic nude [Crl:NU(NCr)-Foxn1nu] (Charles River Laboratories). All tumors were allowed to reach a volume of 50 mm^3^ and mice were sorted into groups with approximately equal tumor volumes before treatment commenced. Mouse sample size was chosen using an alpha set at 0.05 a priori with a power of 80 (n = 6). Mice were dosed via intraperitoneal injection (IP) with either vehicle control (DMSO:Tween80:PBS/10:10:80) or 60 mg/kg SR9243 (Cayman Chemicals) once daily for the duration of the experiment. Tumor volume V was measured via perpendicular measurements using electronic calipers (V = 1/2(L × W^2^)). Tumor volume and weight were monitored once every two days. Mice were monitored daily for signs of illness, pain, or severe weight loss. All mice were humanely euthanized using CO_2_ followed by cervical dislocation. Tumors and livers were weighed. Axillary, brachial, inguinal, and superficial cervical lymph nodes were isolated and stained for flow cytometry analysis.

### CD8 *in vivo* depletion

E0771 (3 × 10^6^) cells were planted subcutaneously into the lower left mammary fat pad of 6-week-old C57BL/6J mice (Jackson Laboratories). All tumors were allowed to reach a volume of 50 mm^3^ and mice were sorted into groups (n = 10) with approximately equal tumor volumes before treatment commenced. Sample size was chosen using an alpha set at 0.05 a priori with a power of 80. Mice were given 3 initial IP injections of 200 µg IgG or anti-CD8 antibody every three days for 1 week followed by a weekly booster dose until the conclusion of the experiment. Tumor bearing mice were dosed via *IP* with either vehicle 60 mg/kg SR9243 once daily for the duration of the experiment. Tumor volume was measured via electronic calipers as above.

### Human samples

Informed consent was obtained from all human subjects prior to PMBC sample collection in strict accordance with the rules and guidelines stipulated by the Saint Louis University Internal Review Board (IRB). All experimental protocols were approved by the Saint Louis University IRB (protocol #10287).

### Human CD8 isolation

PMBC samples were acquired from whole blood from healthy, adult female volunteers. CD8+ T cells were isolated using the RosetteSep Human CD8+ T Cell Enrichment Cocktail (StemCell Technologies). Briefly, whole blood was incubated with the CD8+ T cell enrichment cocktail and then diluted with PBS + 2% FBS (Gibco). Whole blood was separated via density gradient using Lymphoprep in SepMate-50 tubes (StemCell Technologies) via centrifugation at 1200xg for 10 minutes. Supernatants were decanted and washed with PBS + 2% FBS twice via centrifugation at 300xg for 10 minutes. Cells were counted via hemocytometer, resuspended in RPMI 1640 + GlutaMAX (Gibco) supplemented with 10% FBS (Gibco), 1X penicillin, 1X streptomycin (Caisson Labs). Cells were plated in 24 well plates coated with 10 µg/mL anti-CD28 (BioLegend) and 1 µg/mL anti-CD3 (BD Biosciences) at 1 × 10^6^ cells per well in the presence or absence of 5 µg/mL PD-L1 (R&D Systems) and either vehicle (DMSO) or SR9243 (10 µM). Cells were treated for 4–18 hours as stated and then subjected to flow cytometry analysis.

### Single-cell RNA-sequencing data analysis

Published sc-RNA-seq data from immune cells isolated from Blood, Lymph-nodes, Normal and Tumor tissue from 8 patients. This dataset contained two TNBC tumors BC3 and BC5 (deposited at Geo-Ascension Viewer: GSE114727^44^). Samples (~18,000 cells) were examined using Seurat and normalized for high variance/mean gene expression. The matrices produced were then assessed as one Seurat object (scData) using the Intergratedata function in Seurat. Scaling, PCA, UMAP, tSNE reduction and clustering were all implemented in the standard_integration_workflow.R and cluster_exploration.R scripts.

### Bone marrow derived macrophage differentiation

6 to 10-week-old C57/BL6 mice were euthanized using CO_2_ followed by cervical dislocation. Femurs and tibias were isolated. Bone marrow was flushed with sterile PBS and cells were resuspended in RPMI 1640 + GlutaMAX media supplemented with 15% L929 supernatant and plated in petri dishes (Gibco). On day 3, 5 mL media was added to cultures. Cells were isolated from dishes on day 7 and subjected to described experimental conditions.

### CD4 T-cell polarization

Lymphocytes and splenocytes were isolated from 6–10-week-old female C57BL6 mice (Jackson Laboratory). CD4+ T cells were purified using the MACs CD4+ T cell isolation kit (Miltenyi) according to manufacturer’s instructions. Cells were plated (5 × 10^5^ cells per well) in 24 well dishes (Corning) and coated with 1 µg/mL anti-CD3 (BioLegend) and 2 µg/mL anti-CD28 (BD Pharmigen) in RPMI 1640 + GlutaMAX (Gibco) supplemented with 10% HD FBS (Gibco), 10 mM HEPES (VWR), 1 mM sodium pyruvate (Sigma), 50 µM 2-mercaptoethanol (National Diagnostics), 1X penicillin, and 1X streptomycin (Caisson Labs). On Day 2, cells were split into uncoated 24 well plates (Falcon) with fresh supplemented media. On Day 7, cells were harvested for use in experiments as described in the presence of DMSO or SR9243 (10 µM) for 24 h. For quantification of cytokine production (IL2, IFNγ and IL6), differentiated CD4 T cells were stimulated in in 24 well dishes (Corning) coated with 1 µg/mL anti-CD3 (BioLegend) and 2 µg/mL anti-CD28 (BD Pharmigen) in the presence or absence of 50% E0771 conditioned media and E0771 cell lysate and either vehicle control or SR9243 (10 µM) for 24 h.

### Mouse CD8 T- cell polarization

Lymph nodes and spleen were isolated from 6–10-week-old female C57BL6 mice (Jackson Laboratory). CD8a + T cells were purified using the MACs CD8a + T cell isolation kit (Miltenyi) according to manufacturer’s instructions. Cells were plated (5 × 106 cells/well) in 6 well dishes coated with 2.5 µg/mL anti-CD3 (BioLegend) and 5 µg/mL anti-CD28 (BD Pharmigen) in RPMI 1640 + GlutaMAX (Gibco) supplemented with 10% HD FBS (Gibco), 1% insulin-transferrin-sodium selenite (Gibco), 50 µM 2-mercaptoethanol (National Diagnostics), 1X penicillin, and 1X streptomycin (Caisson Labs). On day 1, media was supplemented with 0.5 ng/mL IL-7 (BioLegend) and 20 ng/mL IL-2 (BioLegend). On day 2, cells were split (1 × 106 cells/well) into uncoated 6 well dishes. On day 4, cells were harvested for use and subjected to described experimental conditions.

### Amplex-Red staining

CD8+ T-cells were isolated and differentiated under CD8 polarizing conditions for 3 days as described. Cells were plated on multi-chamber glass slides then treated with 100 nM SR9243 or 100 nM GW3965 for 24 h in the presence of E0771 tumor conditioned media (TCM) or complete E0771 media (RPM1 + Glutamax + 10%FBS). Cells were stained with Amplex-Red according to the manufacture’s instructions and assessed for cholesterol content using purified cholesterol standards and assessed for fluorescent emission detection at ~590 nm with excitation 530–560 nm and microplate reader.

### Cell culture

E0771 cells were purchased from CH3 BioSystems and EMT6 from ATCC. Cells were maintained in RPMI 1640 + GlutaMAX (Gibco) supplemented with 10 mmol/L HEPES (VWR) and 10% FBS (Gibco). Cells were grown at 37 °C in a humidified atmosphere of 5% CO_2_. Cells were split when 70–85% confluent. Conditioned media was collected from confluent cells, filter sterilized and then flash frozen. E0771 cells were harvested with trypsin/EDTA (Caisson), resuspended in 1 mL complete media, and sonicated. Conditioned media and lysate samples were stored at −80 °C until use.

### Cytokine bead array assay

Differentiated CD8 T cells were stimulated in 24 well plates (Corning) coated with 2.5 µg/mL anti-CD3 (BioLegend) and 5 µg/mL anti-CD28 (BD Pharmigen) in the presence or absence of 50% E0771 conditioned media and E0771 cell lysate and either vehicle control or SR9243 (10 µM) for 48 hours. Supernatants were collected and frozen at −20 °C. Cytokine production was analyzed using the Cytometric Bead Array Mouse Th1/Th2/Th17 Cytokine Kit (BD Biosciences) according to manufacturer’s directions. Samples were collected using a BD FACs Canto II (BD Biosciences) and analyzed using FCAP Array Software (BD Biosciences).

### CD8 T-cell cytotoxicity/cell killing assay

Differentiated CD8 T cells were stimulated in 24 well plates (Corning) coated with 2.5 µg/mL anti-CD3 (BioLegend) and 5 µg/mL anti-CD28 (BD Pharmigen) in the presence or absence of 50% E0771 conditioned media and E0771 cell lysate and either vehicle control or SR9243 (10 µM) for 24 hours. Cytotoxicity was assessed using the 7-AAD/CFSE Cell-Mediated Cytoxocity Assay kit (Caymen Chemical). Briefly, E0771 cells were stained with CFSE at 37 °C for 15 minutes. Stained cells were washed with media and incubated at a 1:1 ratio with CD8 T cells for 4 hours at 37 °C. Cells were resuspended in cell-based assay buffer containing 7-AAD for 15 minutes at 4 °C, fixed, and then analyzed via flow cytometer. Flow cytometry data was analyzed using FlowJo V10 software (TreeStar).

### Flow cytometry

Cells were stimulated with 50 ng/mL PMA (Cayman Chemical) and 1 µg/mL ION (Sigma-Aldrich) for 4 hours at 37 °C. After 1-hour GolgiStop (BD Biosciences) was added to all samples. For human T cell studies and CD8 re-stimulation studies, GolgiStop (BD Biosciences) was added to all samples 1 hour prior to staining. Cells were resuspended in cell staining buffer (BioLegend) and stained with FVS780 viability dye (BioLegend), Fc Block (BioLegend), and various extracellular antibodies. Cells were washed in cell staining buffer and fixed using Fixation Buffer (ThermoFisher) for 1 hour at 4 °C. Cells were then washed in Permeabilization Buffer (ThermoFisher) and stained with intracellular antibodies overnight. Cells were washed in Permeabilization Buffer and resuspended in cell staining buffer. All flow cytometry data was collected on LSR II (BD Biosciences) and analyzed using FlowJo V10 software (TreeStar). For every sample 1 × 10^5^ events were uniformly collected for all FACs analyses described.

### Seahorse metabolic profiling

Differentiated CD8+ T-cells were stimulated in 24 well plates (Corning) coated with 2.5 µg/mL anti-CD3 (BioLegend) and 5 µg/mL anti-CD28 (BD Pharmigen) in the presence or absence of 50% E0771 conditioned media and E0771 cell lysate and either vehicle control or SR9243 (10 µM) for 1 hour. Stimulated CD8 T cells were resuspended in Seahorse XF DMEM Base media (Agilent) supplemented with 1mM L-glutamine (Sigma) for glycolytic stress test or 2mM-L-glutamine, 1 mM sodium pyruvate (Sigma), and 2 mM glucose (Sigma) for mitochondrial stress test. Resuspended cells were plated in 96-well microplate (Agilent) at 7.0 × 10^5^ cells per well and were incubated in at 37 °C in a humidified atmosphere without CO_2_ for 1 hour. Wells were injected with 10 mM glucose, 1 mM oligomycin, and 50 mM 2-deoxyglucose obtained from the Glycolytic Stress Test kit (Agilent) or 1 µM oligomycin, 0.5 µM FCCP, and 0.5 µM antimycin A + rotenone obtained from the Mitochondrial Stress Test kit (Agilent). Extracellular acidification rates and oxygen consumption rates were measured using the Seahorse XFe96 Analyzer (Agilent).

### MTS reduction assay

E0771 cells were cultured in 96 well plates and treated with designated amounts of SR9243 for 96 hours in media containing 10% HD FBS. Cell-viability was assessed using the Cell-titre 96 kit (Promega) according to manufacturer’s guidelines.

### Oil-red-o staining

10μm frozen tumor sections were fixed with 4% PFA in PBS for 10 minutes at room temperature. Slides were washed twice with 1X PBS and permeabilized with 1X PBST for 5 minutes at room temperature with gentle rocking. Slides were washed three times with 1X PBS for ten minutes each at room temperature, then stained with Oil Red-O solution (4 g/l ORO powder in 60% isopropanol) at room temperature for 10 minutes. Slides were washed twice with 1X PBS and counterstained with Vectashield with DAPI mounting medium (Vector Labs).

### CFSE CD8+ T-cell polarization assay

Freshly isolated CD8 T cells were labeled with 1 µM CellTrace CFSE (ThermoFisher) for 20 minutes at room temperature. Cells were then differentiated as described and then stained for viability (FVS780), CD4, and CD8. Flow cytometry data was collected on an LSR II (BD Biosciences) and analyzed using FlowJo V10 software (TreeStar).

### Luciferase reporter assays

HEK293 cells were reverse transfected using Lipofectamine 2000 Transfection Reagent (Invitrogen) at 80% confluency and seeded in 96-well plates at 20,000 cells per well. GFP expression assessed via on EVOS FL Auto (Life Technologies) was used to assess transfection efficiency. Forty-eight hours post-transfection cells were treated with either vehicle (DMSO) and LXR synthetic agonist (GW3965) at indicated concentrations or increasing concentrations of either BT549 or E0771 conditioned media. Post 24-hour treatment, cells were lysed, and luciferase activity was measured using the Dual-Glo Luciferase Assay System (Promega). Luminescence was measured using Synergy Neo microplate reader (BioTek). The values were normalized using Renilla expression.

### Quantitative RT-PCR

Differentiated CD8 T cells were stimulated in 24 well plates (Corning) coated with 2.5 µg/mL anti-CD3 (BioLegend) and 5 µg/mL anti-CD28 (BD Pharmigen) in the presence or absence of 50% E0771 conditioned media and E0771 cell lysate and either vehicle control or SR9243 (10 µM) for 48 hours. Total RNA was harvested from cultured cells using the PureLink RNA mini kit according to manufacturer’s instructions (ThermoFisher). Isolated RNA (1 µg) was reverse transcribed into cDNA using the qScript cDNA Synthesis kit according to manufacturer’s instructions (QuantaBio). Quantative PCR (qPCR) was performed with SYBR Select Master Mix (Applied BioSystems) with select cognate primers and on a QuantStudio 6 Flex or QuantStudio 7 Flex (Life Technologies). Gene expression was normalized to cyclophilin (*PP1a*).

### Immunoblotting

Confluent E0771 cells were treated with vehicle control (DMSO), SR9243 (10 µM), or GW3965 (10 µM) for 24 hours. Protein was isolated from cells and quantified using a BCA assay (Thermo Scientific). Protein was boiled at 950 C for 5 minutes in the presence of Lamelli and DTT. Protein samples were run on SDS page gel and transferred to a membrane via Trans-BlotTM Turbo (BioRad). The membrane was washed with TBST and then blocked for 1 hour at room temperature in either 5% BSA or 5% milk. Primary antibody was added and incubated at 40 C overnight. The membrane was then washed and incubated with secondary antibody for 1 hour at room temperature. The membrane was washed and then visualized using ECL (BioRad) in a Chemidoc XRS (BioRad). Images were quantified using ImageJ (version 1.51j8).

### Project funding

This project was partially funded by grants from the Department of Defense (W81XWH-16-1-0333), the National Institutes of Health, the National Cancer Institute (1R21CA205096-01A1) and the Siteman Cancer Center Investment Program.

## Supplementary information

Supplementary Information
