# Supplementary material for: LXR-inverse agonism stimulates immune-mediated tumor destruction by enhancing CD8 T-cell activity in triple negative breast cancer
Source: Sci Rep. 2019 Dec 20;9:19530. doi: 10.1038/s41598-019-56038-1 (PMC6925117; doi:10.1038/s41598-019-56038-1)
Supplement: Supplementary file 1 — Supplementary Information [file 41598_2019_56038_MOESM1_ESM.pdf]

Supplementary Information

LXR-inverse agonism stimulates immune-mediated tumor destruction by enhancing CD8 T-cell activity in triple negative breast cancer.

Authors: Katherine J. Carpenter[1], Aurore-Cecile Valfort[2], Nick Steinauer[1], Arindam Chatterjee[1], Suomia Abuirqeba[1], Shabnam Majidi[1], Monideepa Sengupta[1], Richard J. Di Paolo[3,5], Laurie P. Shornick[4], Jinsong Zhang[1,5], Colin A. Flaveny[1,5]*

[1] The Department of Pharmacology and Physiology, Saint Louis University School of Medicine, Saint Louis MO 63104.

[2] The Center for Clinical Pharmacology, Saint Louis College of Pharmacy, Saint Louis MO 63110.

[3] The Department of Molecular Microbiology and Immunology, Saint Louis University School of Medicine, Saint Louis MO 63104.

[4] The Department of Biology, Saint Louis University, Saint Louis MO 63103

[5] The Alvin J. Siteman Cancer Center at Barnes-Jewish and Washington University School of Medicine in Saint Louis, Saint Louis MO 63110.

Corresponding Author:

Colin A. Flaveny PhD Email: colin.flaveny@health.slu.edu Phone: 314-977-6460

Figure S1

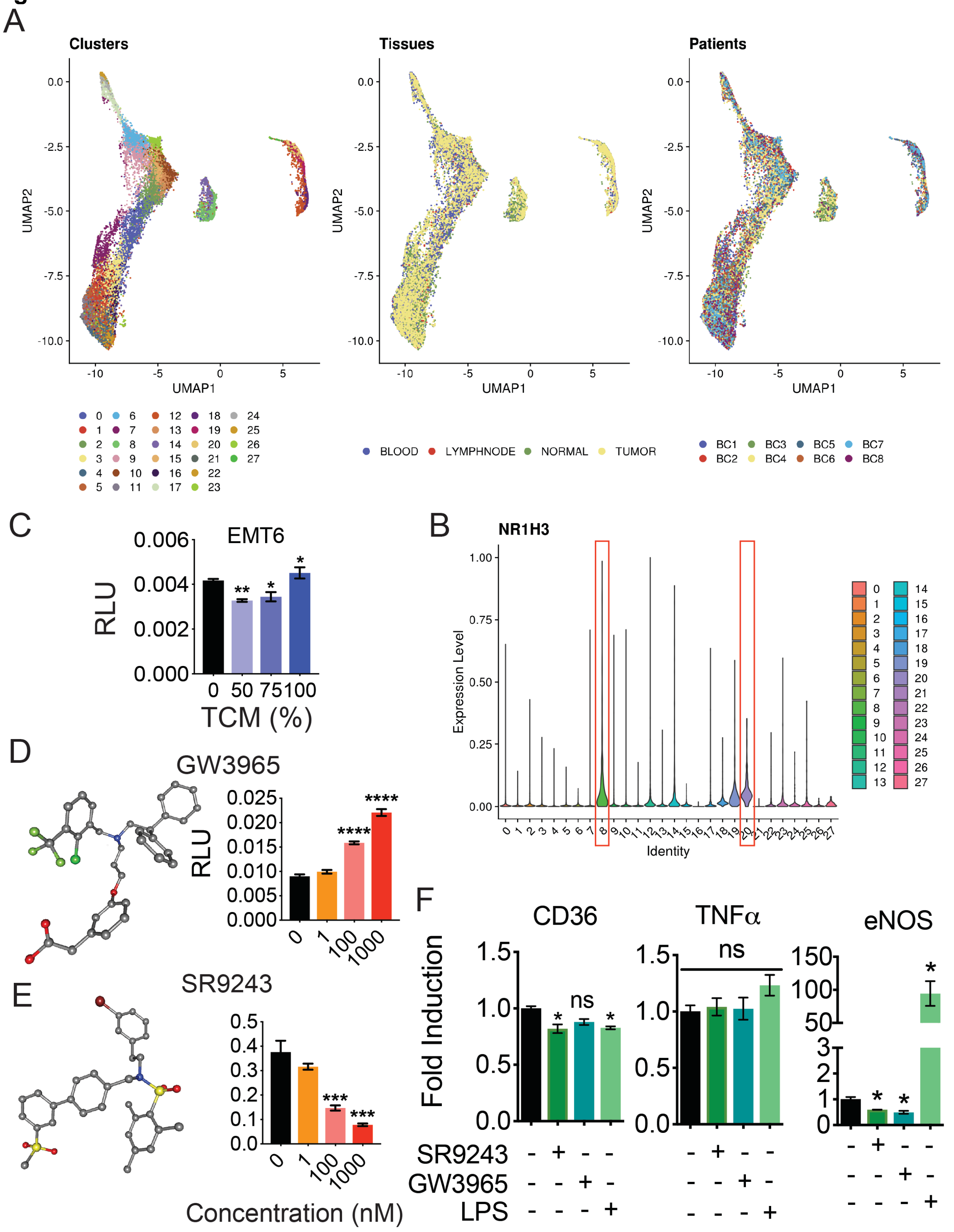

A
Clusters
Tissues
Patients
UMAP1
UMAP2
BLOOD
LYMPHNODE
NORMAL
TUMOR
BC1
BC2
BC3
BC4
BC5
BC6
BC7
BC8
B
NR1H3
Expression Level
Identity
C
EMT6
RLU
TCM (%)
D
GW3965
E
SR9243
Concentration (nM)
F
CD36
TNFα
eNOS
Fold Induction
SR9243
GW3965
LPS

Figure S2

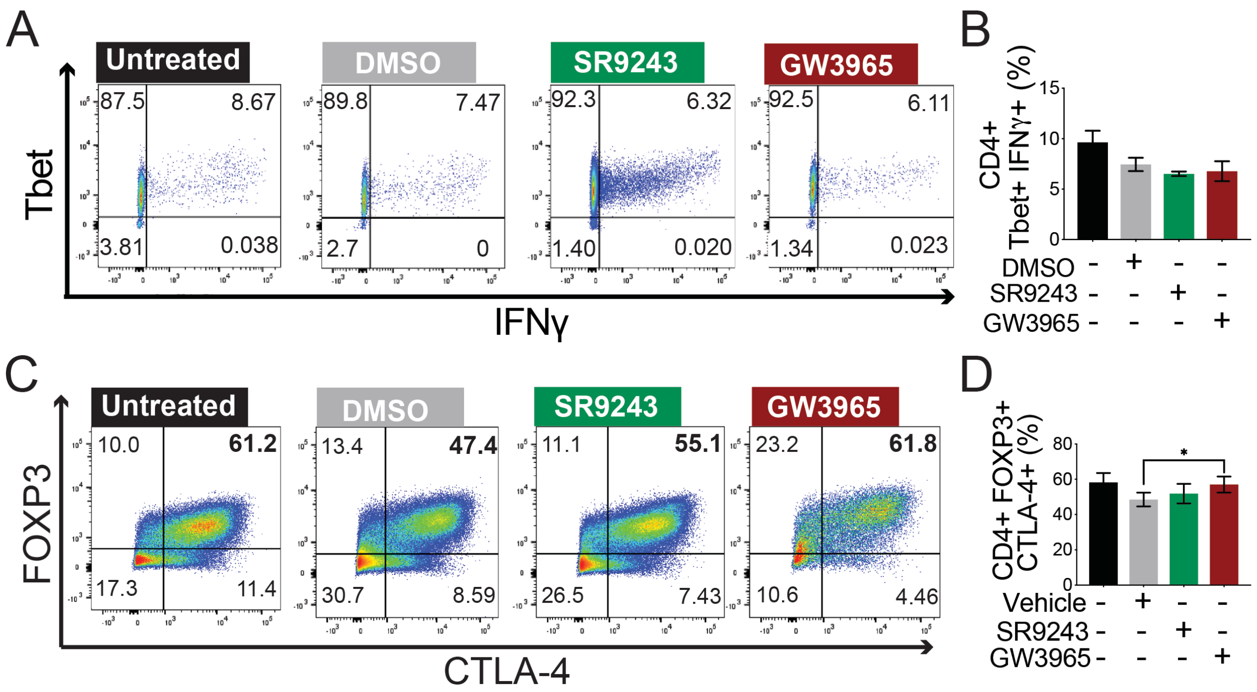
A
Untreated
DMSO
SR9243
GW3965
Tbet
IFNγ
87.5
8.67
3.81
0.038
89.8
7.47
2.7
0
92.3
6.32
1.40
0.020
92.5
6.11
1.34
0.023
B
CD4+ Tbet+ IFNγ+ (%)
DMSO - + - -
SR9243 - - + -
GW3965 - - - +
C
FOXP3
CTLA-4
10.0
61.2
17.3
11.4
13.4
47.4
30.7
8.59
11.1
55.1
26.5
7.43
23.2
61.8
10.6
4.46
D
CD4+ FOXP3+ CTLA-4+ (%)
*
Vehicle - + - -
SR9243 - - + -
GW3965 - - - +

Figure S3

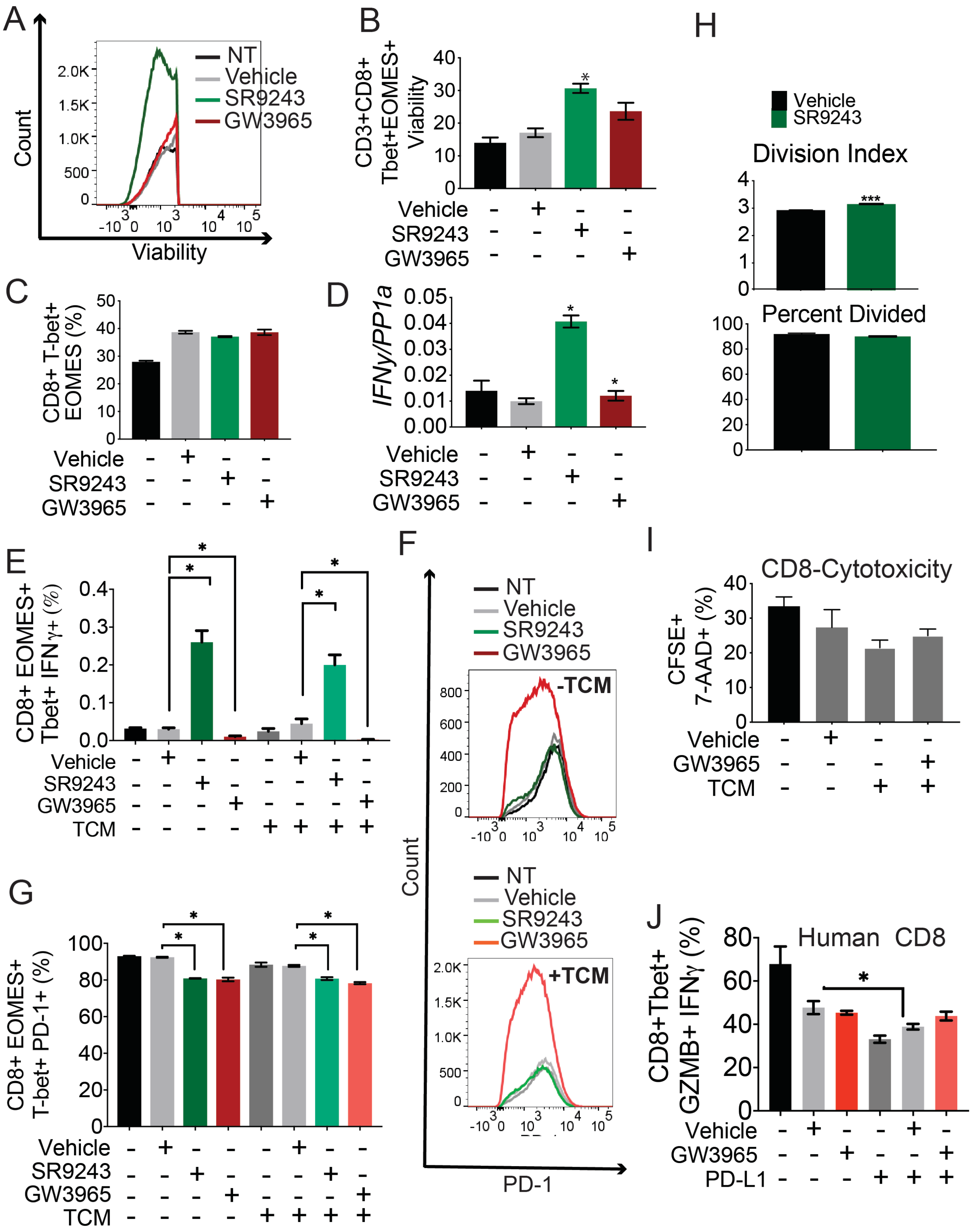

A
NT
Vehicle
SR9243
GW3965
Count
Viability
B
CD3+CD8+ Tbet+EOMES+ Viability
Vehicle
SR9243
GW3965
H
Vehicle
SR9243
Division Index
Percent Divided
C
CD8+ T-bet+ EOMES (%)
D
IFNγ/PP1a
E
CD8+ EOMES+ Tbet+ IFNγ+ (%)
TCM
F
-TCM
+TCM
PD-1
I
CD8-Cytotoxicity
CFSE+ 7-AAD+ (%)
G
CD8+ EOMES+ T-bet+ PD-1+ (%)
J
Human CD8
CD8+Tbet+ GZMB+ IFNγ (%)
PD-L1

Figure S4

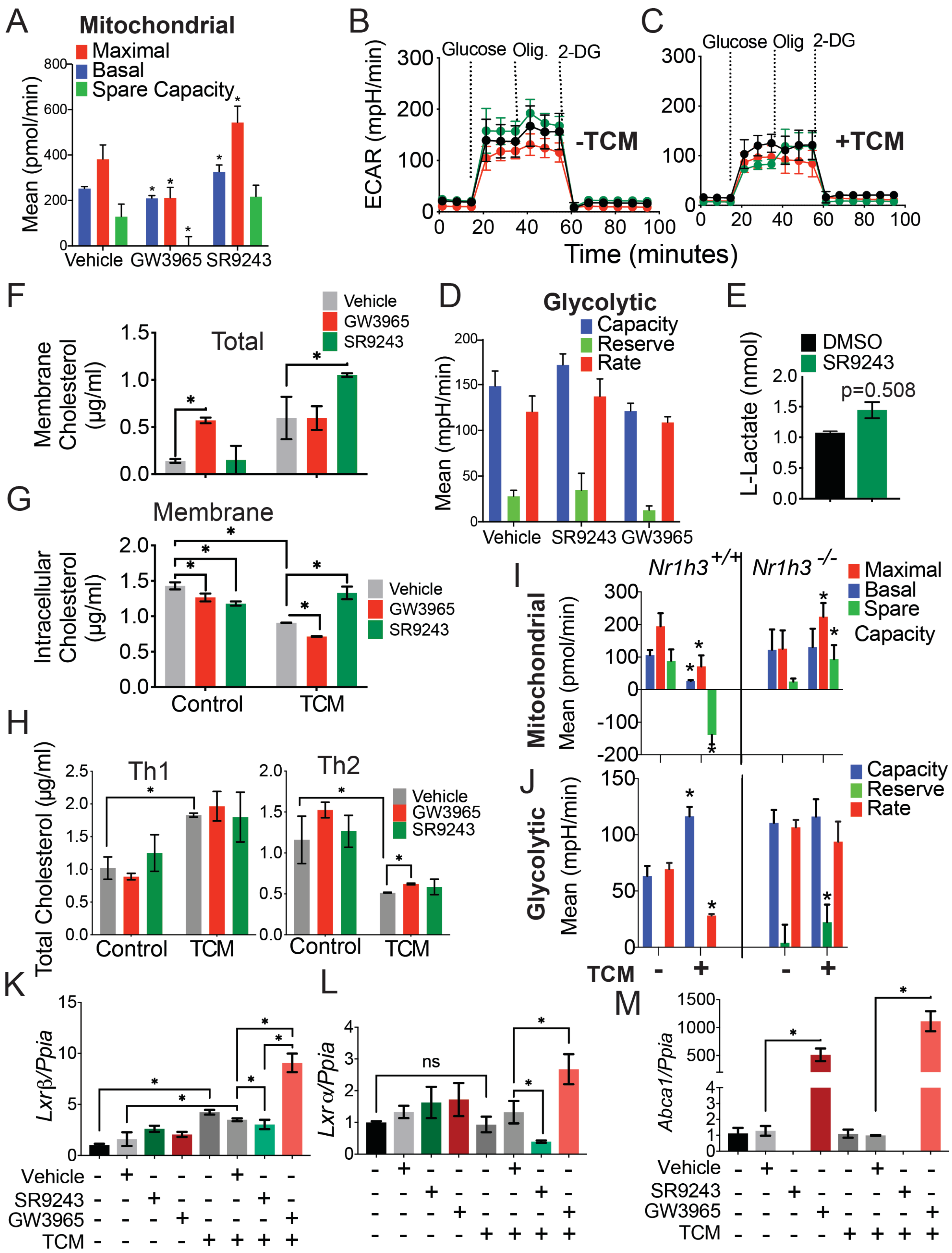
A
Mitochondrial
Maximal
Basal
Spare Capacity
Mean (pmol/min)
Vehicle GW3965 SR9243
B
ECAR (mpH/min)
Glucose Olig. 2-DG
-TCM
C
Glucose Olig 2-DG
+TCM
Time (minutes)
F
Total
Vehicle
GW3965
SR9243
Membrane Cholesterol (µg/ml)
D
Glycolytic
Capacity
Reserve
Rate
Mean (mpH/min)
Vehicle SR9243 GW3965
E
DMSO
SR9243
p=0.508
L-Lactate (nmol)
G
Membrane
Intracellular Cholesterol (µg/ml)
Control
TCM
Vehicle
GW3965
SR9243
H
Th1
Th2
Total Cholesterol (µg/ml)
Control
TCM
Vehicle
GW3965
SR9243
I
Nr1h3 +/+
Nr1h3 -/-
Maximal
Basal
Spare Capacity
Mitochondrial
Mean (pmol/min)
J
Capacity
Reserve
Rate
Glycolytic
Mean (mpH/min)
TCM
K
Lxrβ/Ppia
Vehicle
SR9243
GW3965
TCM
L
Lxrα/Ppia
ns
M
Abca1/Ppia
Vehicle
SR9243
GW3965
TCM

Figure S5

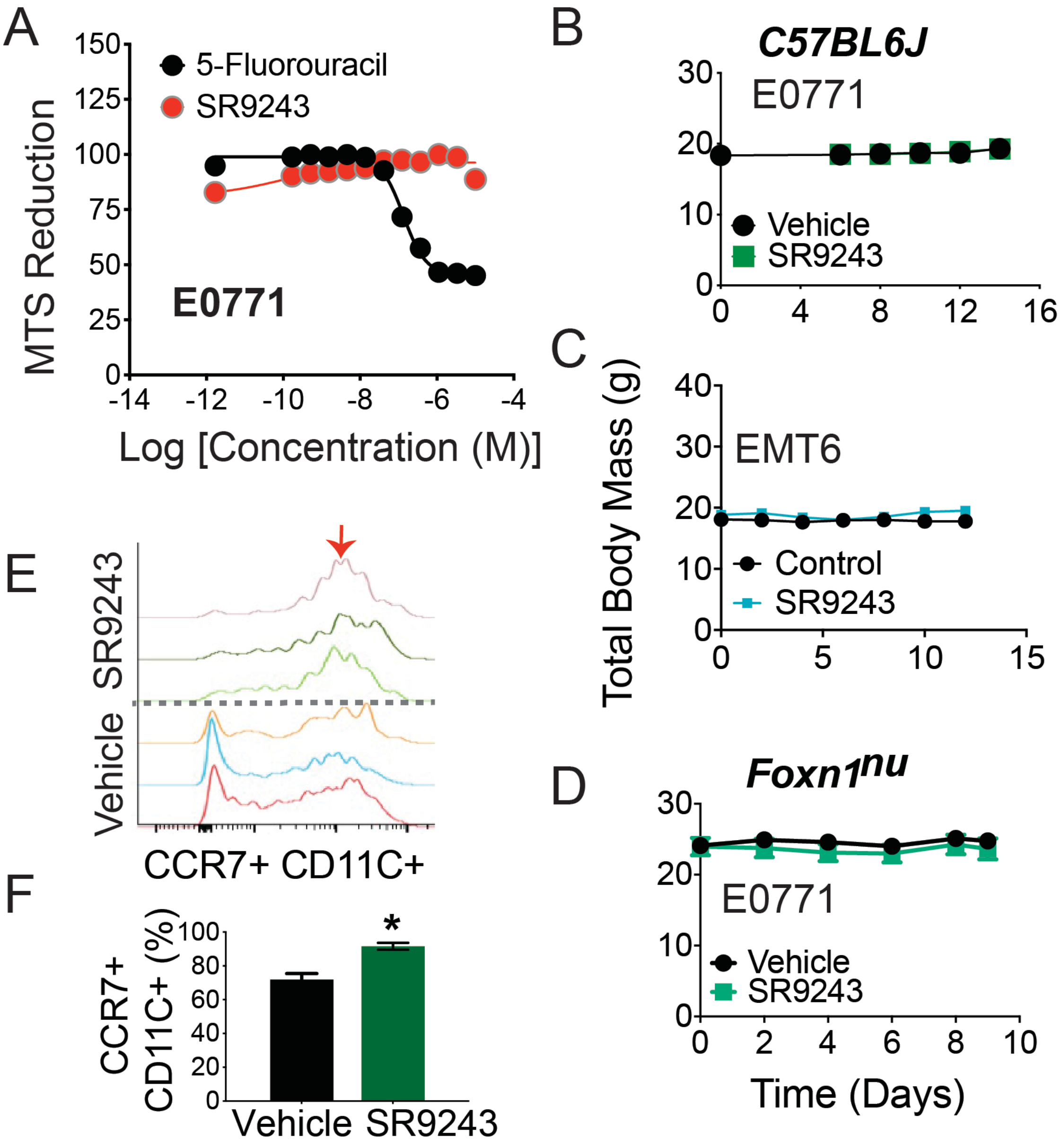
A
150
125
100
75
50
25
0
MTS Reduction
5-Fluorouracil
SR9243
E0771
-14 -12 -10 -8 -6 -4
Log [Concentration (M)]
B
C57BL6J
E0771
30
20
10
0
Vehicle
SR9243
0 4 8 12 16
C
40
30
20
10
0
EMT6
Control
SR9243
0 5 10 15
Total Body Mass (g)
D
Foxn1nu
30
20
10
0
E0771
Vehicle
SR9243
0 2 4 6 8 10
Time (Days)
E
SR9243
Vehicle
CCR7+ CD11C+
F
CCR7+ CD11C+ (%)
100
80
60
40
20
0
*
Vehicle SR9243

Figure S6

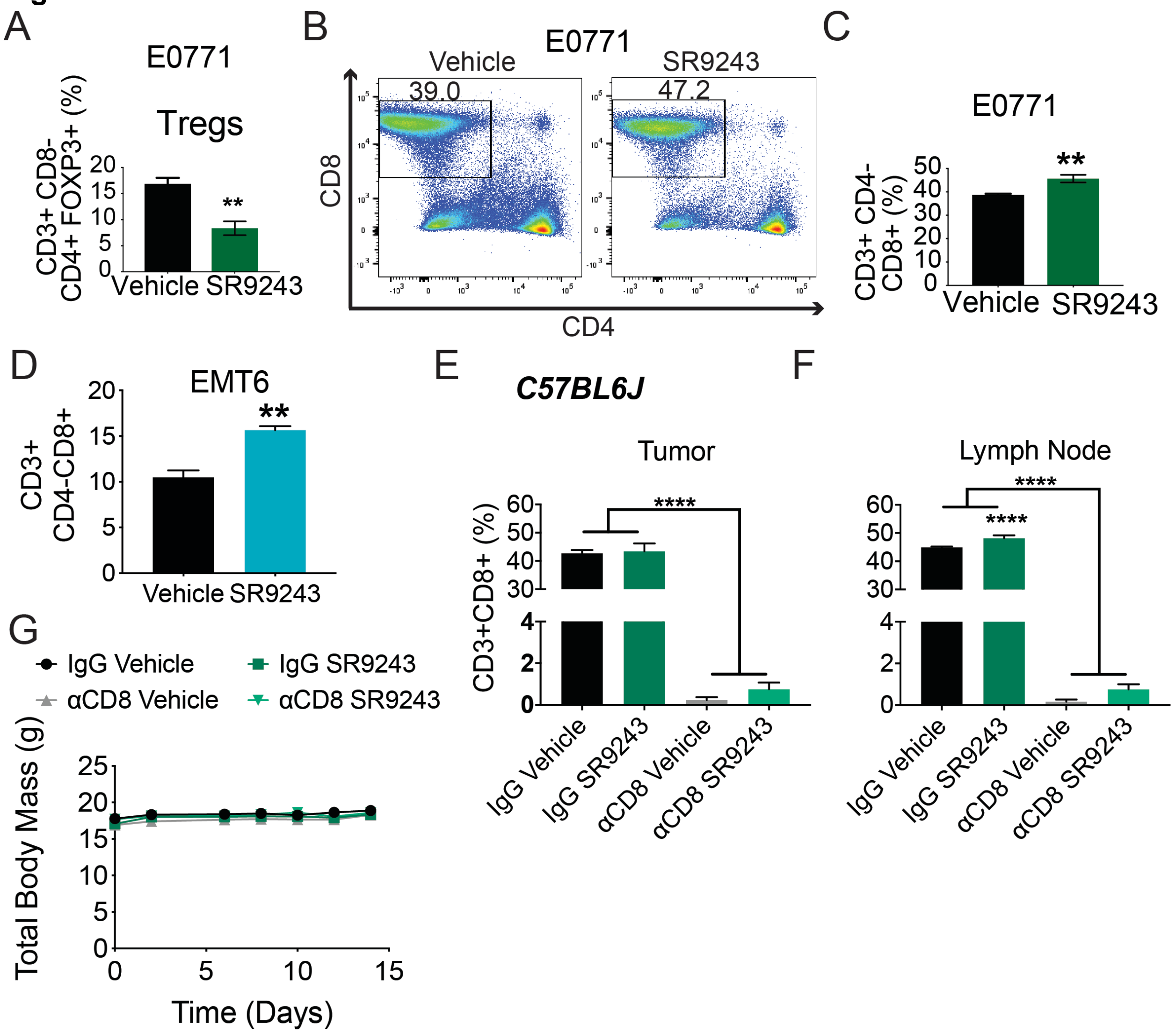
A
E0771
Tregs
CD3+ CD8- CD4+ FOXP3+ (%)
20
15
10
5
0
**
Vehicle SR9243
B
E0771
Vehicle
SR9243
39.0
47.2
CD8
CD4
C
E0771
**
50
40
30
20
10
0
CD3+ CD4- CD8+ (%)
Vehicle SR9243
D
EMT6
20
15
10
5
0
**
CD3+ CD4-CD8+
Vehicle SR9243
E
C57BL6J
Tumor
****
60
50
40
30
4
2
0
CD3+CD8+ (%)
IgG Vehicle
IgG SR9243
αCD8 Vehicle
αCD8 SR9243
F
Lymph Node
****
****
60
50
40
30
4
2
0
IgG Vehicle
IgG SR9243
αCD8 Vehicle
αCD8 SR9243
G
IgG Vehicle
IgG SR9243
αCD8 Vehicle
αCD8 SR9243
Total Body Mass (g)
25
20
15
10
5
0
0
5
10
15
Time (Days)

Supplementary Figure Legends

Figure S1. TNBC cells produce LXR-agonists that inhibit macrophage activation (**A**) FACs dot-plot of sc-RNA-sequencing data showing the distribution clusters based on Left Panel: the 27 unique cell-type clusters identified. Middle Panel: tissue of origin (Blood, Lymphoid, Normal and Tumor). Right Panel: patient tumor (BC1-BC8). Original dataset was previously published by Aziz et al[47]. Cluster analysis performed using Seurat as described in Materials and Methods. (**B**) sc-RNA-seq expression data showing the expression of *NR1H3* in Seurat-identified cell-type clusters. Clusters 8 and 20 correspond to myeloid lineages. (**C**) LXRE-Luc reporter assay showing LXR activation in response to increasing amounts of EMT6 tumor-conditioned media (TCM). (**D**) Structure of the synthetic LXR agonist GW3965. Right Graph: LXRE-Luc reporter assay showing LXR activation in response to increasing amounts of GW3965 (**E**) Structure of the LXR inverse agonist SR9243. Right Graph: LXRE-Luc reporter assay showing LXR activation in response to increasing amounts of SR9243. (**F**) RT-QPCR data showing quantified expression of anti-inflammatory marker CD36, or the pro-inflammatory markers TNFα and eNOS in naïve macrophages treated with 10μM SR9243, 5μM GW3965 for 16 h. *$p<0.05$ determined by 1-Way ANOVA. ns: not significant

Figure S2. SR9243 does not promote CD4+ T-cell activity and GW3965 stimulates Treg CTLA4 expression. (**A**) FACs dot-plot showing percentage of IFNγ producing Th1 polarized in response to SR9243 or GW3965 cells. (**B**) Graph showing the average mean percentage of IFNγ producing cells (n=4) in Th1 cells treated with 10μM SR9243, 5μM GW3965 or DMSO vehicle. (**C**) FACs data showing the stimulation of CTLA4 expression

in Treg cells (CD3+CD4+FOXP3+) (**D**) Graph showing the mean expression of CTLA4 in Tregs treated with GW3965, SR9243 or DMSO control for 24 h. CD4+ (Th0) splenocytes and lymphocytes were isolated from *C57BL6J* mice using a pan CD4+ negative selection affinity column (Miltenyi) and differentiated under Treg polarizing conditions as described. Cells were treated with 10μM SR9243, 5μM GW3965 or DMSO for 7 days or with LXR ligands and either 50% E0771 TCM or RPMI1640 control media for 24 h starting on day 6 of differentiation where stated. FACs histograms are representative results. All bar graphs are averages percentages from three repeat experiments. *p<0.05 1-Way ANOVA. *p<0.05 determined by 1-Way ANOVA

Figure S3. LXR inhibition activates CD8+ T-cells. (A) FACs data showing the effect of LXR ligands on CD8+ effector memory T-cell (CD3+CD8+Tbet+Eomes+) viability. (**B**) Mean percent viability of CD8+ effector memory T-cells exposed to SR9243 or GW3965 (n=4). (**C**) Mean percentage of effector memory T-cells produced in response to LXR ligand exposure. (**D**) QPCR-quantified IFNγ expression in CD3/CD28 stimulated CD8+ T-cells treated with SR9243 or GW3965. (**E**) FACs quantification showing the percentage of IFNγ producing cells that are induced in response to SR9243 or GW3965 in the presence or absence of TCM. (**F**) FACs histogram showing expression of PD-1 in CD8+ effector memory T-cells treated with SR9243 or GW3965. (**G**) Graph summarizing the average PD-1 expression in effector memory CD8 T-cells in response to LXR ligand exposure. Effector memory T-cells (CD3+CD8+Tbet+Eomes+) were cultured in CD8-differentiation media for 3 days then exposed to ligands for 24 h. (**H**) Proliferation analysis showing the Percent Divided and Division Index of CFSE stained CD8+ T-cells in

response to SR9243 or DMSO treatment. CD8+ T-cells were differentiated for 4 days and exposed to LXR ligands for 24 h and stimulated with CD3/CD28 4 h prior to FACs analysis. (**I**) CD8+ T-cell cytotoxicity assay showing E0771 viability in response to CD8+ T-cells treated with GW3965 with or without TCM (**J**) Graph showing the percentage of activated human CD8+ T-cells (CD8+Tbet+GZMB+IFNγ+) produced in response to GW3965 with or without or PD-L1. CD8+ T-cells were isolated from PMBCs from healthy female volunteers via negative selection using the RosetteSep™ Human CD8+ T Cell Enrichment Cocktail. Cells were then exposed to 5μg/mL purified human PD-L1 protein or vehicle (PBS) in the presence of 100nM SR9243 for 24 h*$p<0.05$ ***$p<0.001$ determined by 1-Way ANOVA or student's t-test only two means are compared.

Figure S4. LXR inhibition enhances CD8+ T-cell mitochondrial metabolism. (**A**) Graph of mean oxygen consumption rate (OCR) showing the maximal, basal and spare respiratory capacity of CD8+ T-cells treated with vehicle, 100nM GW3965 or 100nM SR9243. (**B**) Oxygraph showing the extracellular acidification rate (ECAR) of CD8+ T-cells treated with vehicle, 100nM- SR9243 or GW3965. (**C**) Oxygraph showing the extracellular acidification rate (ECAR) of TCM exposed CD8+ T-cells treated with vehicle, 100nM- SR9243 or GW3965. (**D**) Mean ECAR showing the glycolytic capacity, reserve and rate, basal and spare respiratory capacity of CD8+ T-cells treated with vehicle, 100nM GW3965 or 100nM SR9243. (**E**) Quantified L-lactate content in CD8+ T-cells in response to 100nM SR9243. L-lactate were quantified using L-Lactate quantification kit (Abcam) based on manufacturer's instructions. (**F**) Total cholesterol content in CD8+ T-cells treated with SR9243 or GW3965 with or without exposure to E0771-TCM or control

E0771 media. (**G**) Membrane cholesterol content in CD8+ T-cells treated with SR9243 or GW3965 with or without exposure to E0771-TCM or control E0771 media. (**H**) Total cholesterol content in Th1 (CD3+CD4+Tbet+) and Th2 (CD3+CD4+Gata3+) exposed to TCM or control E0771 media. Cells were exposed to LXR ligands and E0771 antigens for 16 h, pelleted and washed with PBS then subjected to the cholesterol content quantification. Cholesterol content was quantified using the Amplex-Red® cholesterol assay kit per the manufacturer's instructions (Thermo-Fisher Scientific). (**I**) Graph of mean oxygen consumption rate (OCR) showing the maximal, basal and spare respiratory capacity of CD8+ T-cells from wildtype or *LXR*α knockout mice exposed to E0771-TCM or control media. (**J**) Mean ECAR showing the glycolytic capacity, reserve and rate of CD8+ T-cells from wildtype or *LXR*α knockout mice exposed to E0771-TCM or control media. (**K-M**) RT-QPCR-quantified expression of (K) *Lxr*α, (L) *Lxr*β and (M) *Abca1* in CD8+ T-cells treated with SR9243 or GW3965 along with cotreatment with 50% E0771-TCM and whole cell lysate or control media (RPMI1640). CD8+ T-cells were exposed to LXR ligands and TCM and lysate for 24 h (n=6). *$p<0.05$ 1-Way ANOVA or students t-test where only 2 means are compared.

Figure S5. SR9243 induces immune mediated TNBC tumor destruction in vivo. (**A**) MTS-reduction assay showing E0771 resistance to increasing doses of SR9243 versus the cytotoxic chemotherapeutic 5-Fluorouracil. (**B**) Total body mass of wildtype E0771 inoculated *C57BL6J* mice treated with vehicle (DMSO:Tween80:PBS/10:10:80) or 60mg/kg SR9243 (see Figure 5A-C). (**C**) Total body mass of *C57BL6J* EMT6-tumor bearing mice treated with vehicle or 60mg/kg SR9243 (see Figure 5D-F) (**D**) Total body

mass of immune compromise *Foxn1^{nu}* tumor bearing mice treated with vehicle or 60mg/kg SR9243 (see Figure 5D-F) (**E**) FACs histogram showing expression of CD11c and CCR7 in lymph-node resident antigen presenting cells (macrophages and DCs) (n=3) in vehicle versus SR9243 treated tumor bearing mice. (**F**) Mean percentage of CCR7+ CD11c+ antigen presenting cells (macrophages and DCs) in the draining lymph nodes of mice receiving SR9243 or vehicle (n=3) as determined by FACs.

Figure S6 SR9243-induced tumor destruction is CD8+ T-cell dependent (**A**) Summary FACs data showing the percentage tumor resident Tregs (CD3+CD4+Fox3+) in vehicle versus SR9243 treated mice. (**B**) FACs dot-plot showing the percentage of CD8+ and CD4+ T-cells in SR9243 or vehicle treated tumors. (**C**) Mean percentage of CD8+ T-cells in vehicle versus SR9243 treated mice (n=4). (**D**) Mean percentage of lymph-node resident CD8+ effector T-cells in EMT6 tumor bearing mice treated by SR9243 or vehicle as determined by FACs (n=4). *p<0.05 as determined by student's t-test. (**E**) FACs analysis of showing the mean number of tumor resident CD8+ T-cells (CD3+CD8+) in mice receiving Rat IgG2A control or CD8-T-cell-depleting antibodies. (**F**) FACs data showing the mean number of lymph-node resident CD8+ T-cells (CD3+CD8+) in mice receiving IgG control or CD8-T-cell-depleting antibodies. (**G**) Total body mass of *C57BL6J*-tumor-bearing mice receiving control Rat-IgG2A or CD8-T-cell-depleting antibodies. *p<0.05 **p<0.01 ****p<0.0001 as determined by 1-Way ANOVA or student's t-test where only two means are compared.

Supplementary Materials and Methods

SR9243 Synthesis

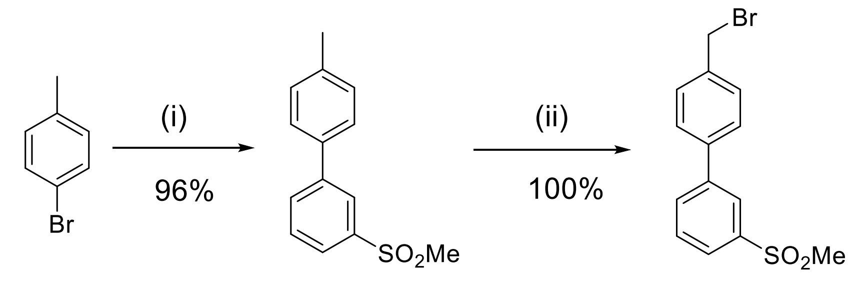

2 **3** **4**

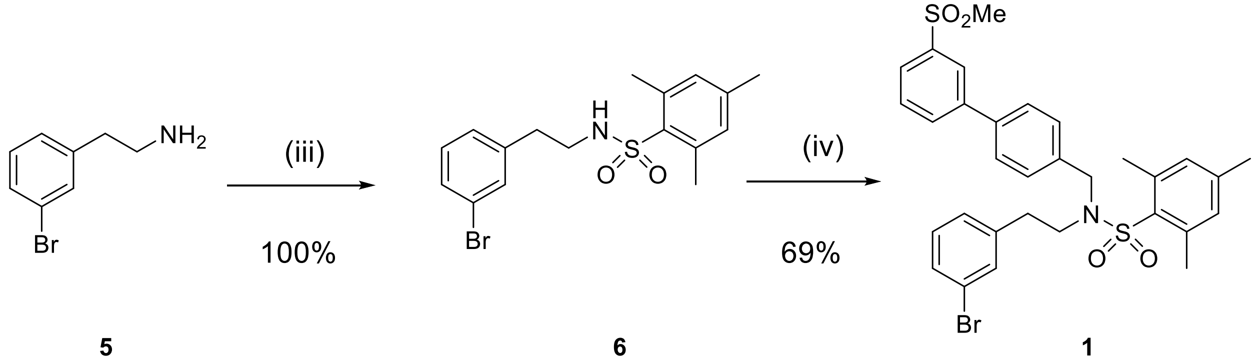

5 **6** **1**

Schematic Representation of SR9243 Synthesis

Reagents and conditions*:* (i) (3-(methylsulfonyl)phenyl)boronic acid, Pd(dppf)Cl_2.CH_2Cl_2, Cs_2CO_3, THF, H_2O, 80°C, 10 h; (ii) *N*-Bromosuccinimide, AIBN (*cat*), DCE, 80°C, 1 h; (iii) 2-Mesitylenesulfonyl chloride, Et_3N, DCM, 10 h; (iv) 4'-(bromomethyl)-3-(methylsulfonyl)-1,1'-biphenyl (**4**), Cs_2CO_3, acetone, 10 h.

Compound **1** was synthesized very efficiently in two steps with an overall yield of 84% from a commercially available starting material (**5**). The synthesis of intermediate benzyl

bromide (**4**) was achieved in high yielding two step sequence from commercially available 4-bromotoluene (**2**).

Experimental

4'-methyl-3-(methylsulfonyl)-1,1'-biphenyl (3): 4-Bromotoluene (**2**) (2g, 11.7mmol), (3-(methylsulfonyl)phenyl)boronic acid (2.6g, 12.9mmol) and cesium carbonate (4.9g, 15.2mmol) were taken in 50mL of 10:1 mixture of THF and H_2O. The reaction mixture was degassed and purged with nitrogen. **[1,1′-Bis(diphenylphosphino)ferrocene]dichloropalladium(II), DCM complex (0.9g, 1.1mmol) was added to it, degassed, purged with nitrogen and heated at** 80°C for 10 h. Reaction mixture was cooled to room temperature, diluted with ethyl acetate, water and the layers separated. The organics were extracted with saturated brine, dried over *anhyd.* sodium sulphate and purified by column chromatography on silica gel using EtOAc-hexane to yield the title compound as a white solid (2.7g, 96%). ^{1}H NMR (400 MHz, DMSO-d_6) □ ppm 2.37 (s, 3 H) 3.29 (s, 3 H) 7.33 (d, *J*=8.40 Hz, 2 H) 7.67 (d, *J*=8.30 Hz, 2 H) 7.73 (t, *J*=7.80 Hz, 1 H) 7.88 (d, *J*=7.80 Hz, 1 H) 8.01 (d, *J*=7.80 Hz, 1 H) 8.13 (s, 1 H). MS (ESI) *m/z* calcd for $C_{14}H_{15}O_2S$ [M+H]$^+$: 247.08. Found: 247.10.

4'-(bromomethyl)-3-(methylsulfonyl)-1,1'-biphenyl (4): 4'-methyl-3-(methylsulfonyl)-1,1'-biphenyl (**3**) (1.36g, 5.51mmol), *N*-Bromosuccinimide (0.98g, 5.51mmol) and azobisisobutyronitrile (0.082g, 0.5mmol) were taken in 50mL of dichloroethane and was heated at 80°C for 1 h under nitrogen. Reaction mixture was cooled to room temperature, diluted with water and the layers separated. The organics were extracted with saturated brine, dried over *anhyd.* sodium sulphate and purified by column chromatography on silica

gel using EtOAc-hexane to yield the title compound as a white solid (1.79g, 100%). ^{1}H NMR (400 MHz, $CDCl_3$-*d*) ppm 3.11 (s, 3 H) 4.56 (s, 2 H) 7.53 (d, *J*=8.50 Hz, 2 H) 7.61 (d, *J*=8.60 Hz, 2 H) 7.67 (t, *J*=7.80 Hz, 1 H) 7.87 (d, *J*=7.80 Hz, 1 H) 7.94 (d, *J*=7.80 Hz, 1 H) 8.16 (s, 1 H). MS (ESI) *m/z* calcd for $C_{14}H_{14}BrO_2S$ $[M+H]^+$: 324.99. Found: 326.90.

***N*-(3-bromophenethyl)-2,4,6-trimethylbenzenesulfonamide (6):** 3-Bromophenethylamine (**5**) (2g, 10mmol) and triehtylamine (2.1mL, 15mmol) were taken in 80mL of dichloromethane (DCE) and stirred for 15min. Mesitylenesulfonyl chloride (2.4g, 11mmol) in 20mL of DCE was added slowly and stirred for 10 h under nitrogen. Reaction mixture was diluted with water and the layers separated. The organics were extracted with saturated brine, dried over *anhyd.* sodium sulphate and purified by column chromatography on silica gel using EtOAc-hexane to yield the title compound as a white solid (3.8, 100%). ^{1}H NMR (400 MHz, $CDCl_3$-*d*) ppm 2.32 (s, 3 H) 2.55 (s, 6 H) 2.73 (t, *J*=6.72 Hz, 2 H) 3.20 (q, *J*=6.60 Hz, 2 H) 4.37 (t, *J*=6.36 Hz, 1 H) 6.96 (s, 2 H) 7.00 - 7.04 (m, 1 H) 7.02 (d, *J*=7.60 Hz, 1 H) 7.12 - 7.17 (m, 2 H) 7.36 (d, *J*=8.00 Hz, 1 H). MS (ESI) *m/z* calcd for $C_{17}H_{21}BrNO_2S$ $[M+H]^+$: 382.05. Found: 382.00.

***N*-(3-bromophenethyl)-2,4,6-trimethyl-N-((3'-(methylsulfonyl)-[1,1'-biphenyl]-4-yl)methyl) benzenesulfonamide (1):** *N*-(3-bromophenethyl)-2,4,6-trimethylbenzenesulfonamide (**6**) (2.12g, 5.55mmol) and cesium carbonate (3.2g, 9.9mmol) were stirred for 30 min in 80mL of acetone. 4'-(bromomethyl)-3-(methylsulfonyl)-1,1'-biphenyl (**4**) (1.8g, 5.55mmol) in 20mL of acetone was added slowly to the mixture and stirred for 10 h under nitrogen. It was then concentrated *in vacuo*,

diluted with DCM and extracted with saturated $NaHCO_3$. The organics were extracted with saturated brine, dried over *anhyd.* Na_2SO_4, filtered, and purified over silica gel using EtOAc-hexane to yield the title compound as a white solid (2.42, 69%). ^{1}H NMR (400 MHz, $CDCl_3$-*d*) □ ppm 2.35 (s, 3 H) 2.63 (s, 6 H) 2.72 (t, *J*=7.70 Hz, 2 H) 3.11 (s, 3 H) 3.32 (t, *J*=7.58 Hz, 2 H) 4.45 (s, 2 H) 6.92 (d, *J*=7.83 Hz, 1 H) 6.98 (s, 2 H) 7.02 - 7.11 (m, 2 H) 7.31 (d, *J*=8.07 Hz, 3 H) 7.59 (d, *J*=8.31 Hz, 2 H) 7.67 (t, *J*=7.80 Hz, 1 H) 7.87 (d, *J*=7.82 Hz, 1 H) 7.93 (d, *J*=7.83 Hz, 1 H) 8.15 (s, 1 H). MS (ESI) *m/z* calcd for $C_{31}H_{33}BrNO_4S_2$ $[M+H]^+$: 626.10. Found: 626.00.
